# Test-Retest Reliability of Short-Interval Intracortical Inhibition Assessed by Threshold-Tracking and Automated Conventional Techniques

**DOI:** 10.1523/ENEURO.0103-21.2021

**Published:** 2021-10-19

**Authors:** Christina S.-Z. Nielsen, Gintaute Samusyte, Kirsten Pugdahl, Jakob U. Blicher, Anders Fuglsang-Frederiksen, Bülent Cengiz, Hatice Tankisi

**Affiliations:** 1 Department of Clinical Neurophysiology, Aarhus University Hospital, Aarhus 8200, Denmark; 2 Department of Clinical Medicine, Faculty of Health, Aarhus University, Aarhus 8200, Denmark; 3 Department of Neurology, Medical Academy, Lithuanian University of Health Sciences, Kaunas 44307, Lithuania; 4 Center of Functionally Integrative Neuroscience, Department of Clinical Medicine, Aarhus University, Aarhus 8200, Denmark; 5 Department of Neurology, Gazi University, Faculty of Medicine, Ankara 06500, Turkey

**Keywords:** automated conventional TMS, short-interval intracortical inhibition, test-retest reliability, threshold-tracking TMS, transcranial magnetic stimulation

## Abstract

Two novel short-interval intracortical inhibition (SICI) protocols, assessing SICI across a range of interstimulus intervals (ISIs) using either parallel threshold-tracking transcranial magnetic stimulation (TT-TMS) or automated conventional TMS (cTMS), were recently introduced. However, the test-retest reliability of these protocols has not been investigated, which is important if they are to be introduced in the clinic. SICI was recorded in 18 healthy subjects using TT-TMS (T-SICI) and cTMS (A-SICI). All subjects were examined at four identical sessions, i.e., morning and afternoon sessions on 2 d, 5–7 d apart. Both SICI protocols were performed twice at each session by the same observer. In one of the sessions, another observer performed additional examinations. Neither intraobserver nor interobserver measures of SICI differed significantly between examinations, except for T-SICI at ISI 3 ms (*p* = 0.00035) and A-SICI at ISI 2.5 ms (*p* = 0.0103). Intraday reliability was poor-to-good for A-SICI and moderate-to-good for T-SICI. Interday and interobserver reliabilities of T-SICI and A-SICI were moderate-to-good. Although between-subject variation constituted most of the total variation, SICI repeatability in an individual subject was poor. The two SICI protocols showed no considerable systematic bias across sessions and had a comparable test-retest reliability profile. Findings from the present study suggest that both SICI protocols may be reliably and reproducibly employed in research studies, but should be used with caution for individual decision-making in clinical settings. Studies exploring reliability in patient cohorts are warranted to investigate the clinical utility of these two SICI protocols.

## Significance Statement

Threshold-tracking short-interval intracortical inhibition (T-SICI) measured with threshold-tracking transcranial magnetic stimulation (TT-TMS) was introduced two decades ago (Fisher et al., 2002). Earlier studies have shown that T-SICI may be used for diagnosing ALS (Vucic and Kiernan, 2006, 2008). However, limitations of the existing serial T-SICI protocol was recently reported and two novel SICI protocols, with potential experimental and clinical utility, were introduced (Samusyte et al., 2018; Tankisi et al., 2021a). These studies were conducted on healthy subjects. The test-retest reliability of the aforementioned protocols was investigated over four identical sessions on 2 days, 5–7 days apart. These results suggest that the two SICI protocols may be reliably and reproducibly employed in research studies with healthy subjects.

## Introduction

Conventional transcranial magnetic stimulation (cTMS) uses magnetic stimulation to measure cortical excitability by applying constant stimulus intensities. If stimulation is applied over the motor cortex, a motor evoked potential (MEP) can be recorded (Kujirai et al., 1993). Cortical excitability can then be measured as changes in averaged MEP (Kujirai et al., 1993).

Threshold-tracking TMS (TT-TMS) is an unconventional TMS method, which also measures cortical excitability (Fisher et al., 2002). Contrary to cTMS, the MEP amplitude is predefined and kept constant by adjusting the stimulus intensities, thus enabling continuous tracking of the motor thresholds and allowing for fluctuations in cortical excitability (Fisher et al., 2002). The method was introduced to counteract restrictions in cortical excitability fluctuations and MEP variability (Fisher et al., 2002; Groppa et al., 2012).

Short-interval intracortical inhibition (SICI) measures cortical inhibition and is a TMS protocol in which two stimuli are delivered with an interstimulus interval (ISI) of 1–7 ms (Kujirai et al., 1993). The first subthreshold stimulus (conditioning stimulus, CS) is followed by a second suprathreshold stimulus (test stimulus; Kujirai et al., 1993), which in TT-TMS is continuously adjusted based on the recorded MEP amplitude (Fisher et al., 2002). In conventional amplitude SICI (A-SICI), cortical inhibition is measured as the relative change in MEP amplitude (Kujirai et al., 1993). In T-SICI (SICI measured by TT-TMS), cortical inhibition is measured as the relative change in stimulus intensity (Fisher et al., 2002).

The precise physiological mechanisms behind SICI are unknown, but SICI at an ISI of 1 ms (SICI_1ms_) is thought to reflect neuronal refractoriness or extrasynaptic GABA-A signaling (Fisher et al., 2002; Roshan et al., 2003; Stagg et al., 2011), whereas SICI at an ISI of 2.5 ms (SICI_2.5ms_), and an ISI of 3 ms (SICI_3ms_) are thought to reflect synaptic GABA-Aergic inhibition (Ziemann et al., 1996; Vucic and Kiernan, 2006). Earlier studies have shown that T-SICI may be used for diagnosing ALS and has been suggested as a biomarker (Vucic and Kiernan, 2006, 2008; Menon et al., 2015; Vucic and Rutkove, 2018). These studies applied a protocol of serial tracking that estimated T-SICI at successively increasing ISIs (Vucic and Kiernan, 2006; Vucic et al., 2006; Matamala et al., 2018). A slightly different tracking strategy was applied in a recent study, in which comparability and reliability of T-SICI and automated A-SICI were explored at ISI 2.5 ms at four different CS intensities in healthy subjects (Samusyte et al., 2018). The CS intensities were tracked in parallel in a pseudorandomized order, a commonly used approach in cTMS (Samusyte et al., 2018). A good correlation of SICI obtained by the two techniques was found across the whole range of CS (Samusyte et al., 2018).

More recently, a good correlation of automated A-SICI and T-SICI at a single CS intensity and ISIs of 1–7 ms with a parallel tracking strategy and important limitations of serial tracking were demonstrated in healthy subjects (Tankisi et al., 2021a). It was proposed that because of a smaller between-subject variability among healthy individuals, A-SICI may be better at demonstrating a pathologic loss of inhibition, which has been observed as an early feature of motor neuron disease (Vucic and Kiernan, 2006). A recent study has demonstrated that both techniques performed well at discriminating ALS patients from patient controls with T-SICI being most reduced before the upper motor neuron signs become apparent (Tankisi et al., 2021b). However, none of the studies assessed the test-retest reliability of the methods, which is important if an investigation is to be used for diagnostic purposes or interventional studies. A trend for improved reproducibility of T-SICI_2.5ms_ has been reported (Samusyte et al., 2018), but it remains unclear whether this applies to other SICI ISIs. Further studies are needed to explore the utility of T-SICI and A-SICI in diagnostic decision-making in ALS, but the comparison of these two methods’ reproducibility and reliability in healthy subjects should be investigated before implementation in clinics. Moreover, T-SICI and A-SICI may potentially investigate different motor neuron pools (Samusyte et al., 2018), and may therefore supplement each other in diagnostics and intervention studies. Therefore, the present study aimed to explore the repeatability and observer reproducibility of the two novel automated conventional and threshold-tracking SICI protocols across ISIs 1–7 ms in healthy subjects. The study also aimed to assess intraday and interday reliability in relation to diurnal variations, which may affect the reliability of SICI measurements (Matamala et al., 2018). No previous study has examined these parameters of T-SICI parallel and automated A-SICI on such an extensive scale.

## Materials and Methods

### Subjects

The study was conducted at Department of Clinical Neurophysiology, Aarhus University Hospital, Denmark, from February 2018 to August 2018. Inclusion criteria were: age above 18 years; absence of neurologic or psychiatric disorders. Exclusion criteria were: pregnancy; use of medication known to affect the nervous system; metal implants. All participating subjects were screened using a modified TMS safety questionnaire (Rossi et al., 2011) and a gross neurologic examination.

Twenty healthy subjects (nine females) were recruited. One subject (S5) was excluded because of undetectable MEP, another subject (S7) because of inability to relax the hand muscles. Eighteen subjects (eight females, mean age: 56.9 years, SD: 12.3; range: 41–77 years) were recruited. One subject (S4, male, age: 46 years) completed all nine TT-TMS examinations, but only seven cTMS examinations by observer 1 because of time restraint. The subject was excluded from the analysis of reliability of cTMS data.

Written informed consent was obtained from all subjects in accordance with the Declaration of Helsinki II. The project was approved by The Central Denmark Region Committees on Health Research Ethics (case: 1-10-72-201-17) and the Danish Data Protection Agency.

### Study design

**Figure 1.**
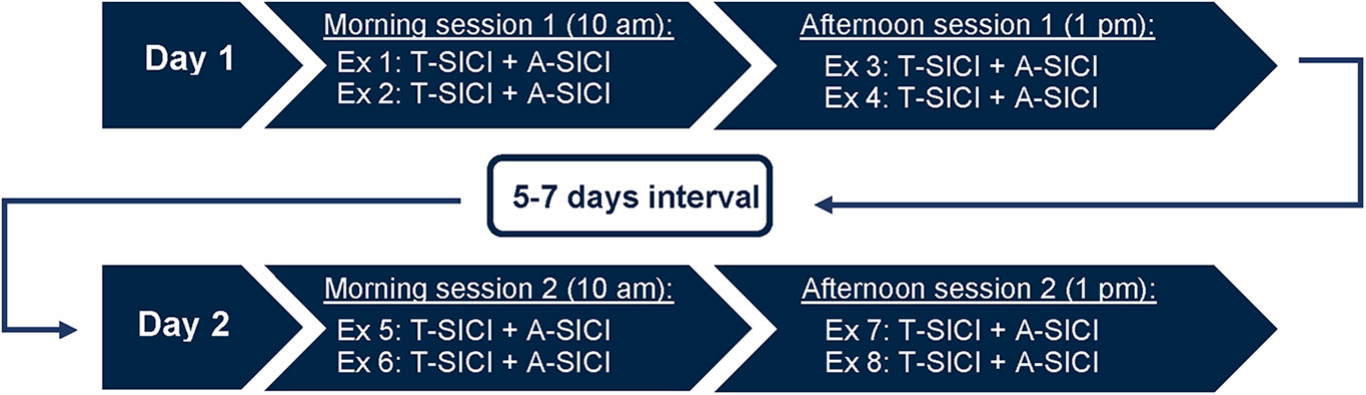
Study design. Each subject was examined on four sessions on two separate days, 5–7 d apart: two sessions in the mornings and two sessions in the afternoons. A-SICI and T-SICI protocols were repeated twice in each session. These examinations were performed by the same observer (observer 1). Two TMS examinations (one A-SICI examination and one T-SICI examination) were additionally performed by a second observer (observer 2) on each subject. The examinations were done in continuation of one of the sessions. In total, nine A-SICI and nine T-SICI examinations were performed on each subject.

To investigate intraobserver test-retest reliability, each subject was investigated by the same observer (observer 1) on two separate days, 5–7 d apart. On the first examination day, two sessions were conducted: a morning session (10.00–11.30 A.M.) and an afternoon session (1– 2:30 P.M.). On the second examination day, a morning session (10–11:30 A.M.) and an afternoon session (1–2:30 P.M.) were conducted again (Fig. 1). Each session consisted of four TMS examinations: two A-SICI examinations and two T-SICI examinations. On each examination day, each subject underwent four A-SICI examinations and four T-SICI examinations. Thus, eight A-SICI examinations and eight T-SICI examinations were conducted on each subject in total by observer 1 (Fig. 1).

All examinations were executed at the same time of day for each subject. Each examination lasted on average 15 min, giving approximately 1 h in total for each session.

To investigate interobserver reliability and reproducibility, a second observer (observer 2) performed an additional TT-TMS and cTMS examination on each subject in continuation of one of the recording sessions. Interobserver (observer 2) examinations were measured either at the morning session of day 1 (*n* = 3), the afternoon session of day 1 (*n* = 7), the morning session of day 2 (*n* = 4) or the afternoon session of day 2 (*n* = 4) because of practical limitations.

Subjects were instructed to restrain from coffee (12 h), alcohol (24 h), and exhaustive exercise (48 h) before TMS examination.

### Experimental setup

The subjects were comfortably seated during the examinations, with their right arm resting in a relaxed position on a pillow placed on their lap. The subjects were instructed to stay relaxed but vigilant. MEP responses were recorded from the relaxed right first dorsal interosseous (FDI) muscle of the right hand using Ag/AgCl ECG electrodes (Ambu WhiteSensor 40713). The active electrode was placed on the belly of the FDI muscle, the reference electrode on the second metacarpophalangeal joint. The ground electrode was placed on the dorsum of the hand. Skin temperature of the subject’s right hand was measured before and after each examination, and a constant temperature was ensured with a heating lamp throughout the examination.

The EMG signal was amplified (1000× gain) and filtered (3–3000 Hz) using a two-channel isolated amplifier (D440-2, Digitimer Ltd.). To remove 50/60-Hz noise, Humbug Noise Eliminator (Digitimer Ltd.) was used as well as a 2000 VA medical transformer (IMEDe 2000, Noratel). To digitize amplified signals, a NI USB-6251 data acquisition system (eight inputs, 16-bit, 1.25 MS/s, National Instruments) was used.

### TMS

Cortical function was assessed using a 70-mm figure-of-eight coil (D70 Remote Coil, reference number 3190-00) connected to two Magstim 200^2^ stimulators in Bistim mode (Magstim Co Ltd). Posterior-anterior current flow in the subject’s motor cortex was induced by placing the coil over the left hemisphere with the coil handle angled 45° postero-laterally to the midsagittal line.

The hand motor hotspot was located by moving the coil in anterior-posterior and medial-lateral directions to induce a MEP of 0.2 mV using a minimal stimulus intensity. Once located, coil-positioning over the hand motor hotspot was kept constant by drawing the coil outline onto a swimming cap worn by the subject. This procedure was repeated before each examination. A spring balancer (SiraFlex, type B) helped steadying the coil. Stimulation frequency was 0.2 Hz.

Automated stimulator control, stimulus delivery, data acquisition and calculation of TMS parameters were managed by the computer software QTRACW (Institute of Neurology, University College London, United Kingdom, distributed by Digitimer Ltd.) using bespoke recording protocols (QTMS-2017).

### Resting motor threshold (RMT)

RMT estimates cortical excitability by measuring the lowest stimulation intensity required to elicit a predefined target MEP. The RMT is estimated before initiation of the SICI protocol and is used as a baseline for calculating CS intensities in SICI. In TT-TMS, the RMT is continuously estimated, “tracked,” during the paired-pulse protocol, as opposed to automated cTMS.

**Figure 2.**
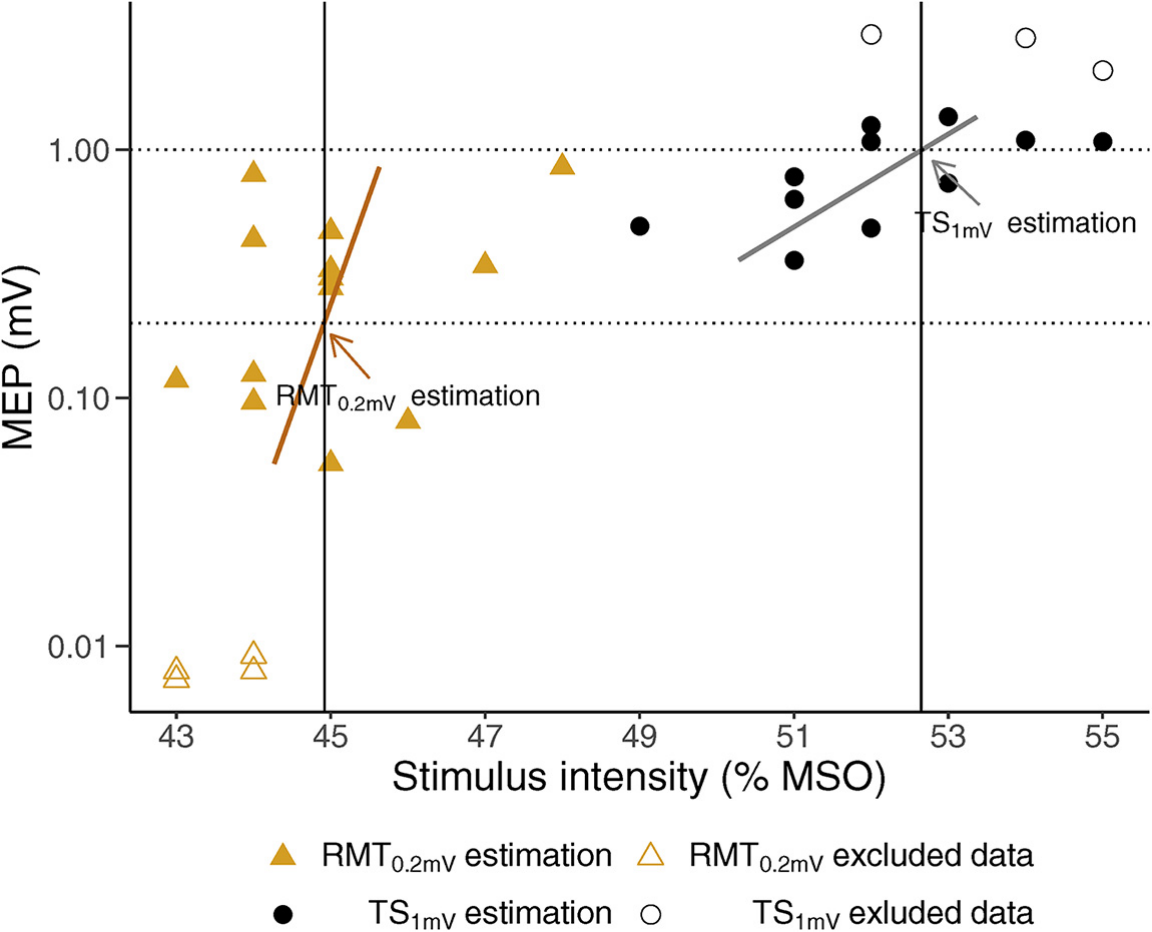
Estimation of RMT_0.2mV_ and TS_1mV_. Scatter plots of recorded MEP against stimulus intensity (in % MSO). RMT_0.2mV_ is the test stimulus (in % MSO) required to evoke a peak-to-peak MEP of 0.2 mV, whereas TS_1mV_ is the test stimulus (in % MSO) required to evoke a peak-to-peak MEP of 1 mV. The figure conceptually shows how RMT_0.2mV_ and TS_1mV_ are estimated. The MEPs were recorded during the RMT_0.2mV_ (orange triangle) and TS_1mV_ (black circle) estimation, before initiation of SICI protocols. RMT_0.2mV_ and TS_1mV_ estimation (solid black vertical lines) were calculated from the intersect of target MEP (dotted horizontal lines) and weighted semi-logarithmic regressions (orange and gray lines), shown by arrows in the figure. Low (<0.02 mV) or high (>2 mV) MEP responses (open circles and open triangles) were excluded from the calculation.

After localization of the motor hotspot, but before SICI protocol initiation, the lowest stimulus intensities (measured in percentage of maximum stimulator output; % MSO) required for eliciting a peak-to-peak target MEP of 0.2 mV (RMT_0.2mV_) and a peak-to-peak target MEP of 1 mV (TS_1mV_) were estimated by threshold-tracking (Fig. 2). The size of the MEPs was analyzed online by QTRACW, which then automatically adjusted the Magstim 200^2^ stimulator output. A proportional tracking mode with a maximum step of 2% MSO was used: the stimulation intensities were adjusted depending on the percentage error of a single MEP (decreased, increased or unchanged if the MEP was above, below, or on target, respectively). A 20% tracking error (on a logarithmic scale) was allowed, and the threshold estimate was considered valid if the MEP hit or bracketed the target line. The RMT_0.2mV_ and TS_1mV_ tracking was deemed stable when six valid estimates had been obtained. RMT_0.2mV_ and TS_1mV_ were automatically calculated by applying a weighted logarithmic regression (Fig. 2). This approach is based on work by Fisher et al. (2002), who found that the stimulus-response curve between the stimulus intensity for single pulse TMS and the MEP amplitude was approximately exponential over a 100-fold range of responses. When plotted on a logarithmic scale, the relationship between stimulus intensity and MEP amplitude was approximately linear in the interval from 0.02 to 2 mV. Target MEP was set at 0.2 mV, the midpoint in this log-linear interval (Fisher et al., 2002). Furthermore, the MEP distributions are often skewed (Nielsen, 1996), and the variability in MEP size, which could represent excitability fluctuations in cortical pyramidal cells and spinal motor neurons, may differ across stimulus intensities (Kiers et al., 1993). Thus, logarithmic transformation of amplitude data has been proposed to ensure normal distributions and to at least somewhat reduce the variability spread across the stimulus intensities (Nielsen, 1996; Goetz et al., 2014). Vucic et al. (2006) deduced that by setting a target MEP located in the midpoint of the log-linear interval, the variability in MEP amplitude translates to smaller changes in stimulus intensity, potentially overcoming these limitations.

**Figure 3.**
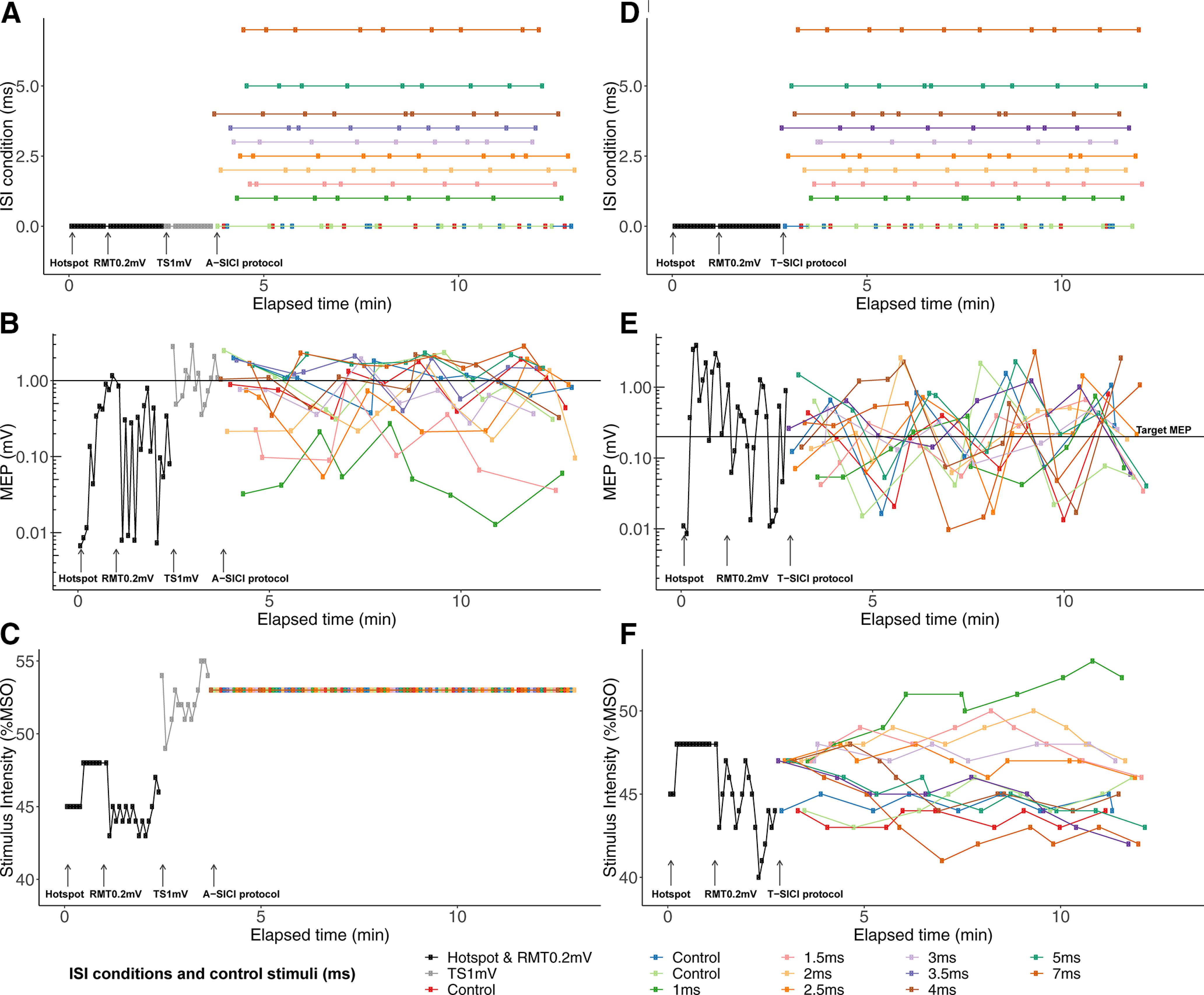
Example recording of A-SICI and T-SICI. Example recording of A-SICI recorded with cTMS (***A–C***) and T-SICI recorded with TT-TMS (***D–F***) for subject 1 of the first examination in morning session 1 from QTRACW. Each color depicts either a different ISI condition (ISI = 1–7 ms) or a control stimulus (ISI = 0 ms). After location of the motor hotspot, RMT_0.2mV_ and TS_1mV_ were estimated, and the A-SICI protocol was initiated (***A–C***). Likewise, after RMT_0.2mV_ estimation, the T-SICI protocol was initiated (***D–F***). For T-SICI, the nine different conditions and control stimuli (***D***) were pseudorandomized and tracked in parallel (***E***). Paired stimulus intensities were adjusted continuously to obtain a target MEP of 0.2 mV (***F***). Likewise, for A-SICI nine different conditions and control stimuli (***A***) were pseudorandomized (***B***). However, the paired stimulus intensities were kept constant for A-SICI (***C***).

The tracked RMT_0.2mV_ estimate was then used to set the CS for both T-SICI and A-SICI protocols to ensure a comparable intensity between the techniques. The SICI protocol was then initiated (Fig. 3).

### T-SICI protocol

The T-SICI protocol was initiated after RMT_0.2mV_ estimation and was executed by QTRACW. The conditioning and test stimulus pairs were given at nine different conditions, with each condition comprising of a different ISI. The ISIs were 1, 1.5, 2, 2.5, 3, 3.5, 4, 5, and 7 ms (Fig. 3*D–F*). For adequate monitoring of the control condition, RMT_0.2mV_ was continuously tracked on three independent channels using 1% MSO steps. The control and paired stimuli were delivered in a pseudorandomized order (Fig. 3*D*). The protocol stopped after each of the nine ISI conditions had been delivered ten times (giving 90 paired stimuli), and RMT_0.2mV_ estimation had been conducted 30 times (giving 30 single stimuli).

The CS and the test stimulus were adjusted continuously. The CS intensity was set to 70% of RMT_0.2mV_. Stimulus adjustments were based on changes in the average RMT_0.2mV_ estimate obtained from three control channels after each pseudorandomization cycle. The test stimulus intensity was initially set to 120% of RMT_0.2mV_. It was then adjusted continuously using a step size of 1% MSO (doubled if two in a row stimuli “missed” the target), to maintain a target MEP of 0.2 mV (Fisher et al., 2002). This was done independently for each SICI ISI condition, resulting in nine conditioned thresholds. To calculate the estimates of control and conditioned thresholds, the recorded log MEP response was plotted against the corresponding test stimulus intensity (Fisher et al., 2002). The test stimulus corresponding to the target MEP of 0.2 mV was then estimated by regression analysis (Fisher et al., 2002). The regression analysis was weighted to account for the position of the data points, so that data points lying closer to the estimated regression line contributed more. Data points lying outside the linear part [0.02–2 mV] of the semi-logarithmic regression were excluded (Fisher et al., 2002). T-SICIs were calculated as T-SICI = (conditioned threshold – RMT_0.2mV_)/RMT_0.2mV_ × 100% (Fisher et al., 2002). Negative values reflect cortical facilitation, and positive values reflect cortical inhibition. Variables of interest were RMT_0.2mV_, T-SICI_1ms_, T-SICI_2.5ms_, T-SICI_3ms_, T-SICI averaged at ISI from 1 to 3.5 ms (T-SICI_1-3.5ms_) and T-SICI averaged at ISI from 1 to 7 ms (T-SICI_1-7ms_). RMT_0.2mV_ was chosen as it reflects cortical excitability threshold. T-SICI_1-3.5ms_ and T-SICI_1-7ms_ were investigated as predictors of general cortical inhibition.

### A-SICI protocol

The A-SICI protocol was also managed by QTRACW and was initiated after RMT_0.2mV_ and TS_1mV_ had been estimated at the beginning of the examination (Fig. 3*A–C*). The same ISI conditions as in the T-SICI protocol were used. The same procedure for pseudorandomization of the nine SICI and three control conditions as in the T-SICI protocol was applied (Fig. 3*A*). The A-SICI protocol also stopped after each of the nine ISI conditions had been delivered ten times, and control stimuli at TS_1mV_ 30 times.

As opposed to the continuous RMT_0.2mV_ estimation and continuous adjustment of the paired stimulus intensities at each ISI condition in T-SICI, in A-SICI the paired stimulus intensities for each condition remained fixed throughout the examination (Fig. 3*C*). The CS intensity was fixed at 70% of the RMT_0.2mV_, and the test stimulus intensity was fixed at TS_1mV_.

Geometric means of MEPs were calculated for each paired stimulus condition (conditioned MEP) and each control stimulus (control MEP). The A-SICIs were calculated as A-SICI = (conditioned MEP/control MEP) × 100% (Kujirai et al., 1993). Values above 100% reflect cortical facilitation, and values below 100% reflect cortical inhibition. The same variables of interest (RMT_0.2mV_, A-SICI_1ms_, A-SICI_2.5ms_, A-SICI_3ms_, A-SICI_1-3.5ms_, and A-SICI_1-7ms_) were chosen as well as TS_1mV_.

### Exclusion of spontaneous muscle contraction

For both protocols, the online gating threshold during recording was set to 15 μV to exclude traces with contamination from spontaneous muscle contraction.

To exclude responses when the target muscle was not completely relaxed, online gating of prestimulus activation was used in both protocols. Sweeps in which negative EMG peaks exceeding 0.015 mV were detected 270 ms before the magnetic stimuli were automatically discarded from the analysis.

### Statistical analysis

Microsoft Excel version 16.16.15. was used for calculation of repeated measures ANOVA (rmANOVA) and Student’s paired *t* test. Other statistical analyses were performed using the statistical software program R version 3.6.2 (2019-12-12) and OriginPro 2017 (OriginLab).

Normality was checked by quantile-quantile plots (QQ-plots) and histograms, and log-normally distributed data were log10-transformed.

Simple linear regression and calculation of correlation coefficients were applied for intraobserver method correlation analysis (Bland and Altman, 2003). RMT_0.2mV_ and SICI variables for each subject were calculated by averaging, either arithmetically (for normally distributed data) or geometrically (for log-normally distributed data), all measurements across all sessions. One sample *t* test comparing to a control condition (0% RMT_0.2mV_ for T-SICI, 100% test MEP for A-SICI) was used to determine significant inhibition or facilitation at each ISI. The relationship between two SICI methods across all ISIs was described by fitting a linear curve [y = a + b × log(10), where a = intercept, b = slope, x = mean group A-SICI, non-transformed].

Intraobserver repeatability was assessed by coefficient of repeatability (CR; Bartlett and Frost, 2008), intraobserver reliability was assessed by intraclass correlation coefficient (ICC; Koo and Li, 2016), and reproducibility of intrasession and intersession measurements were assessed by rmANOVA (Bland and Altman, 1999; Bland, 2015). An overview of the statistical terms is summarized in Table 1. Bland–Altman plots were constructed to examine for systematic observer bias (Bland and Altman, 1999; Bland, 2015). CR was defined as CR = $1.96*\sqrt{2\sigma_{w}}$ (Bland and Altman, 1986, 1999), and 95% confidence interval (CI) were calculated (Barnhart and Barboriak, 2009).

**Table 1:** Overview of definitions, applicability and calculations of repeatability, reliability, and reproducibility

Parameter	Definition	When to use	How to calculate
Repeatability	Variation in repeat measurements made on the same subject under identical conditions: Same method, same observer, measurements are taken in quick succession (Bartlett and Frost, 2008). Variation is ascribed to errors in the measurement process (Bartlett and Frost, 2008; Bland and Altman, 1999).	The CR can be used to study measurement pre*cis*ion (Bartlett and Frost, 2008). It is used when de*cis*ions are made on an individual basis. CR indicates how much two or more measurements made on the same subject will vary on 95% of occasions (Bartlett and Frost, 2008). Thus, the higher the measurement error, the higher the CR.	CR = $1.96*\sqrt{2\sigma_{w}}$, where $\sigma_{w}$ is within-subject variance (Bartlett and Frost, 2008).
			
Reliability	Ratio of the subject variation compared with the total variation: subject variation and measurement error (variation in the measurement process; Bartlett and Frost, 2008). A reliability of 1 indicates no measurement error and 0 indicates that all variation stems from measurement error (Koo and Li, 2016).	The ICC can be used to study the amount of measurement error in measurements made on the same subjects by different observers (interobserver reliability) or by a single observer (intraobserver reliability; Bartlett and Frost, 2008). ICC measures how well subjects maintain their position within the group with repeated measurements (Streiner and Norman, 2008). This is important for sample size and power calculations in interventional studies (Fleiss, 1999; Brown et al., 2017) and provides some indication on a discriminative value of a test (de Vet et al., 2006). As reliability ICC is a dimensionless ratio, ICC can be used to compare methods, whose measurements are on different scales (Koo and Li, 2016).	(SD of subjects’ true values)^2^/[(SD of subjects’ true values)^2^ + (SD measurement error)^2^] (Bartlett and Frost, 2008).
			
Reproducibility	Variation in measurements made on the same subject under changing conditions: Different methods or instruments, different observers, measurements being made at different timepoints, within which the “true” underlying variable could undergo non-negligible changes (Bartlett and Frost, 2008).	Reproducibility can be studied when measurements are made by different observers, with different methods or instruments, or at different timepoints (Bartlett and Frost, 2008). Different statistical analysis methods have different assumptions. Choice of statistical analysis depends on study design, measurement scale, etc.	rmANOVA was used to study difference in timepoints. Paired *t* test (incl. correction for multiple comparison) was used to study interobserver differences.

For normally distributed data, CR and Bland–Altman plots can be interpreted as the *absolute* difference between any two future replicate measurements estimated to be no greater than CR on 95% of occasions. However, this is not the case for CRs and Bland–Altman plots, which have been calculated with log-transformed data. Log-transformed CRs and Bland–Altman plots become dimensionless ratios on back-transformation (Bland and Altman, 2003), and can be interpreted as a *relative* difference between any two future replicate measurements estimated to be no greater than CR on 95% of occasions. A two-way random effects model with single-ratings, absolute-agreement [ICC (2,1)] was applied to quantify ICC (Fleiss, 1999; de Vet et al., 2006; Streiner and Norman, 2008; Koo and Li, 2016; Brown et al., 2017). Reliability was defined as poor (ICC < 0.50), moderate (ICC 0.50–0.749), good (ICC 0.75–0.90) or excellent (ICC > 0.90; Koo and Li, 2016).rmANOVA was also used to estimate between-subject and within-subject variance. If assumption of sphericity for rmANOVA was violated (Mauchly’s sphericity test, *p* < 0.05), Greenhouse–Geisser correction was applied. If significant effects were identified (*p* < 0.05), pairwise *post hoc* analysis with Bonferroni correction for multiple comparisons was applied.

Interobserver statistical parameters were calculated by applying the first examination by observer 1 in the corresponding session. If significant effects were identified (*p* < 0.05), pairwise *post hoc* analysis with Bonferroni correction for multiple comparisons was applied. Interobserver reliability was estimated by ICC. Student’s *t* test for paired data were applied for comparison of interobserver measurements. 95% CI for interobserver estimates are not given, because two observers are too few to give useful estimates.

## Results

### Data characteristics

**Figure 4.**
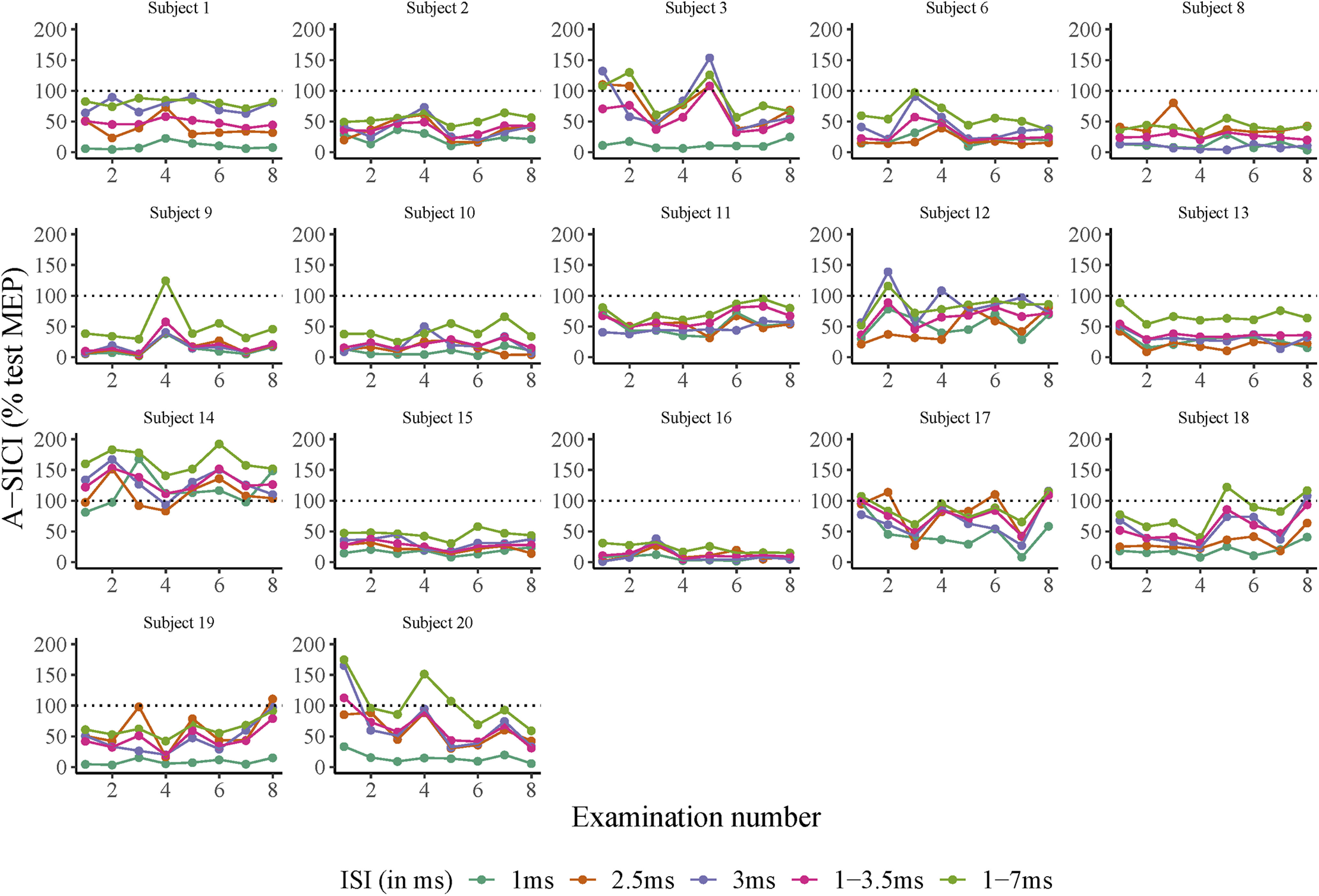
Intraobserver A-SICI examinations for all subjects. *x*-axis: examination number. *y*-axis: SICI values measured with cTMS. Horizontal dotted line denotes threshold for cortical facilitation (A-SICI > 100, in % MEP) or inhibition (A-SICI < 100, in % test MEP). ISI of 1, 2.5, 3, 1–3.5, and 1–7 ms are depicted in different colors. Each subfigure denotes measurements from one individual subject. Subject number 4 (missing data point), subject number 5 (undetectable MEP), and subject number 7 (unrelaxed hand muscles) were excluded.

**Figure 5.**
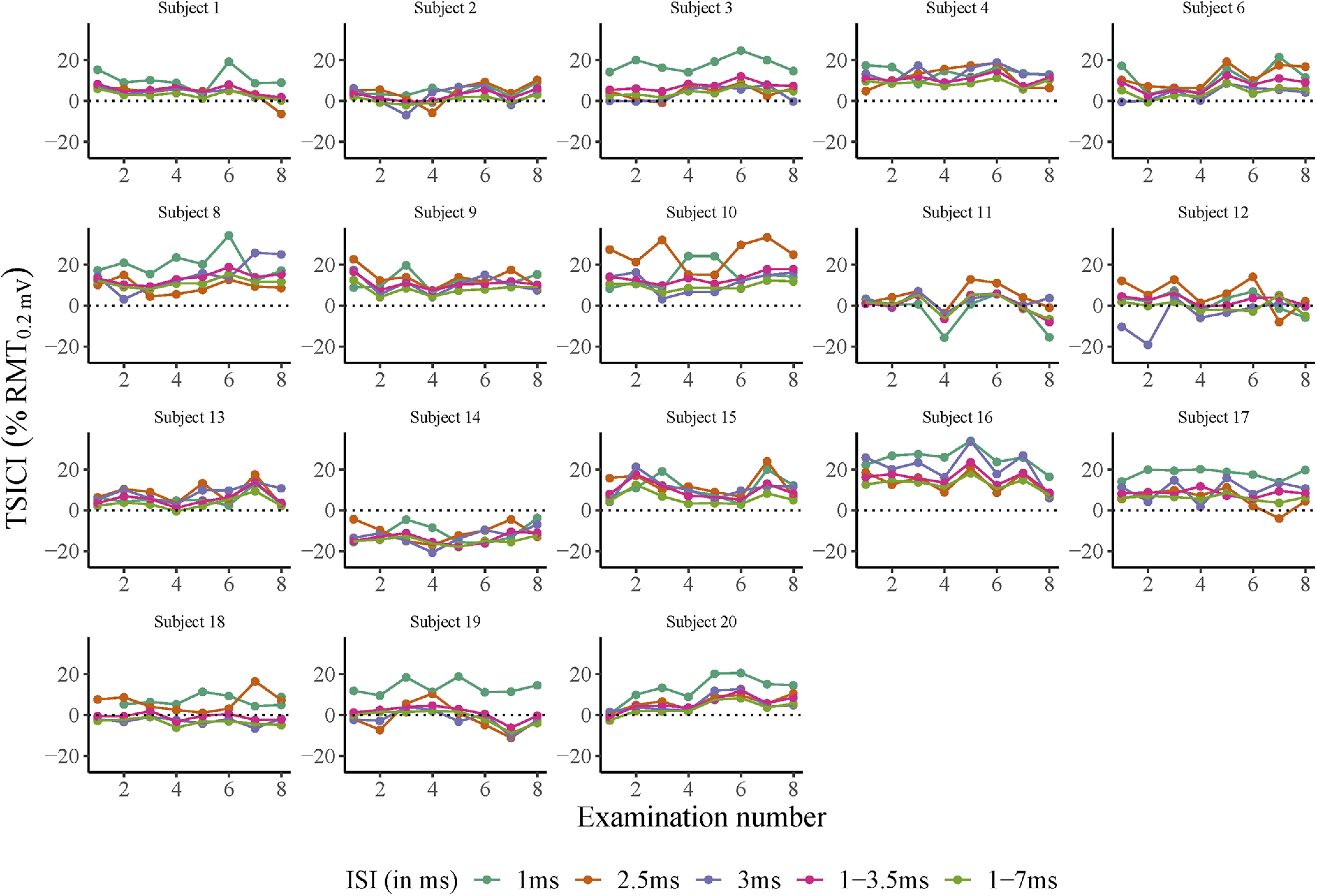
Intraobserver T-SICI examinations for all subjects. *x*-axis: examination number. *y*-axis: SICI measured with TT-TMS. Horizontal dotted line denotes threshold for cortical facilitation (T-SICI < 0, in % RMT_0.2mV_) or inhibition (T-SICI > 0, in % RMT_0.2mV_). ISI of 1, 2.5, 3, 1–3.5, and 1–7 ms are depicted in different colors. Each subfigure denotes measurements from one individual subject. Subject number 5 (undetectable MEP) and subject number 7 (unrelaxed hand muscles) were excluded.

The cTMS and TT-TMS RMT_0.2mV_ were in general below 60% MSO, except for subjects S8 and S11. TS_1mV_ was on average 119.3% (range 109.9–144.7%) of RMT_0.2mV_ for cTMS. Most of the subjects exhibited cortical inhibition at SICI_1ms_, SICI_2.5ms_ and SICI_3ms_ with cTMS and TT-TMS (Figs. 4, 5). Subject S14 (male, age: 75 years) exhibited cortical facilitation with both TMS methods throughout all sessions at SICI_1ms_, SICI_2.5ms_, and SICI_3ms_. Average skin temperature for both methods was 35.4°C [range 34.5–36.4°C]. A-SICIs were log-normally distributed. All other TMS parameters were normally distributed. Description of data are given in Table 2.

**Table 2:** Description of intraobserver measurements

Method	Parameter	Sample size	Total number of measurements	Median [IQR]^a^	Mean	±SE^b^	SD
cTMS	A-SICI_1ms_	17	136	16.1 [16.4]			
	A-SICI_2.5ms_	17	136	37.3 [33.2]			
	A-SICI_3ms_	17	136	40.7 [31.5]			
	A-SICI_1-3.5ms_	17	816	34.5 [26.9]			
	A-SICI_1-7ms_	17	1224	50.9 [32.2]			
	RMT_0.2mV_	17	136		54.2	(±1.94)	8.0
	TS_1mV_	17	136		64.7	(±2.88)	11.9
TT- TMS	T-SICI_1ms_	18	144		10.3	(±2.03)	8.6
	T-SICI_2.5ms_	18	144		7.5	(±1.72)	7.3
	T-SICI_3ms_	18	144		5.5	(±1.89)	8.0
	T-SICI_1-3.5ms_	18	864		5.9	(±1.60)	6.8
	T-SICI_1-7ms_	18	1296		3.5	(±1.51)	6.4
	RMT_0.2mV_	18	144		55.2	(±1.98)	8.4

Measurements of TMS parameters by observer 1. Sample size *n* = 18 for TT-TMS and *n* = 17 for cTMS. Each subject was examined eight times by each method. One subject (S4) was examined seven times with cTMS and eight times with TT-TMS. SICI was measured with the A-SICI and the T-SICI parallel protocol. Only values measured at ISIs 1 ms (SICI1ms), 2.5 ms (SICI2.5ms), 3 ms (SICI3ms), 1–3.5 ms (SICI1–3.5ms), and 1–7 ms (SICI1–7ms) are depicted. Each TMS parameter was measured twice in each session.

a Point estimates for each subject were calculated as geometric means of their measurements. Data are displayed as medians with interquartile ranges [IQR] of subjects’ point estimates.

b Normally distributed data for each subject was arithmetically averaged to calculate group mean (±SE) and SD.

### Method correlation (intraobserver measurements)

**Figure 6.**
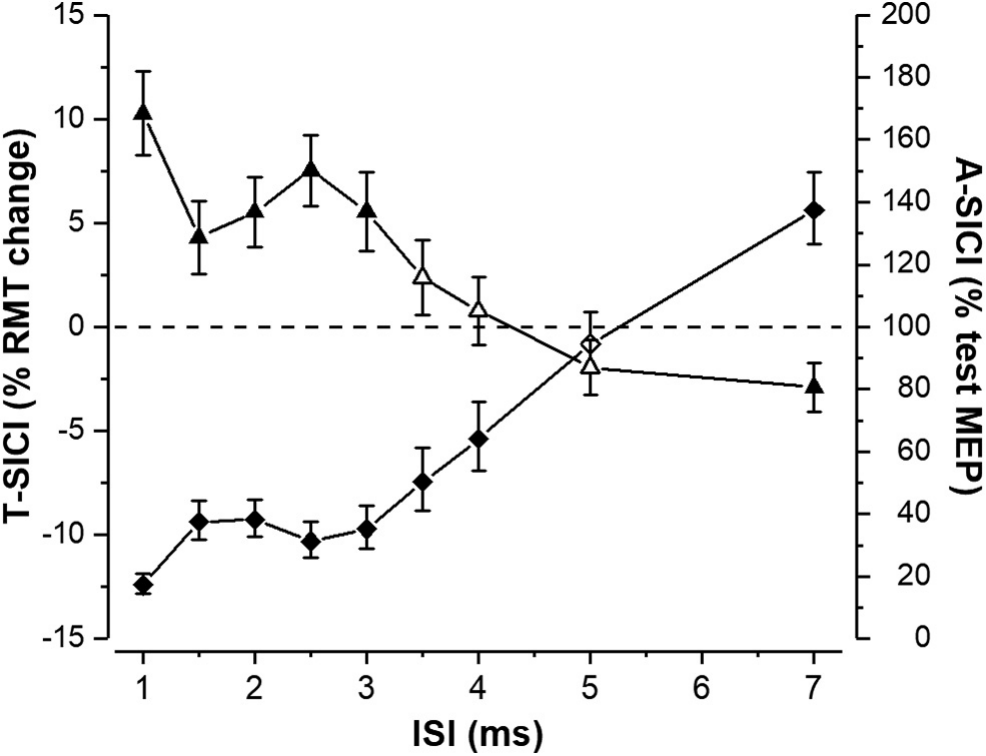
Mean group TT-TMS and cTMS SICI curves. Group means of T-SICI (arithmetic, depicted by triangles) and A-SICI (geometric, depicted by diamonds) were calculated by averaging all 18 individual subjects’ means across all measurements. Error bars represent SEM (±SEM for T-SICI, ×/÷SEM for A-SICI). Filled symbols represent a significant inhibition or facilitation when compared with the control condition (0% RMT_0.2mV_ for TT-TMS and 100% test MEP for cTMS; one-sample *t* test *p* < 0.05).

On a group level, significant inhibition was seen at ISIs 1–3 ms with TT-TMS and at ISIs 1–4 ms with cTMS, with two distinct peaks observed at 1 and 2.5 ms with both techniques (Fig. 6). Meanwhile, there was significant facilitation at ISI 7 ms with both methods.

**Figure 7.**
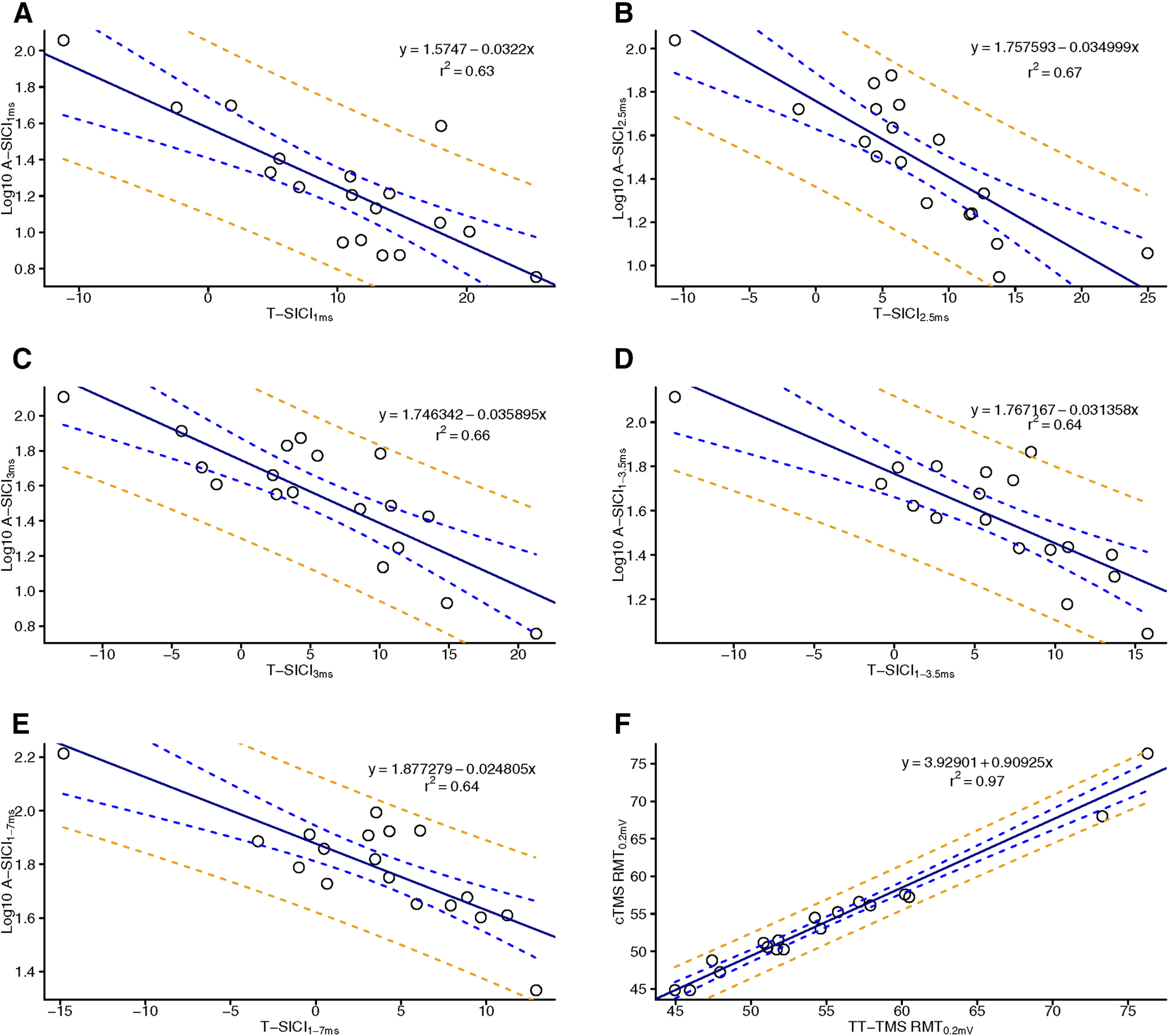
Regression of cTMS and TT-TMS. Simple regression of intraobserver (observer 1) cTMS and TT-TMS parameters. *x*-axis: T-SICI measured with TT-TMS. *y*-axis: Log10 A-SICI measured with cTMS. Regressions depict SICI ISIs measured at 1 ms (***A***), 2.5 ms (***B***), 3 ms (***C***), 1–3.5 ms (***D***), and 1–7 ms (***E***). RMT_0.2mV_ measured with TT-TMS and cTMS (***F***). Full line (black) denotes simple linear regression line, blue dashed line denotes 95% CI for regression line, and orange dashed line denotes 95% prediction intervals for the regression line. Measurements from all eight examinations for all subjects for each TMS parameter were averaged. The TMS regression *r*^2^ are denoted in the upper right corner. All cTMS and TT-TMS measurements correlated significantly.

At individual or averaged ISIs, both intraobserver RMT_0.2mV_ and SICI measurements obtained with cTMS and TT-TMS all correlated significantly (Fig. 7). The correlation coefficient, *r*, for SICIs ranged from 0.79 (95% CI [0.52–0.92]) to 0.82 (95% CI [0.56–0.93]) and was 0.98 (95% CI [0.96–0.99]) for RMT_0.2mV_.

**Figure 8.**
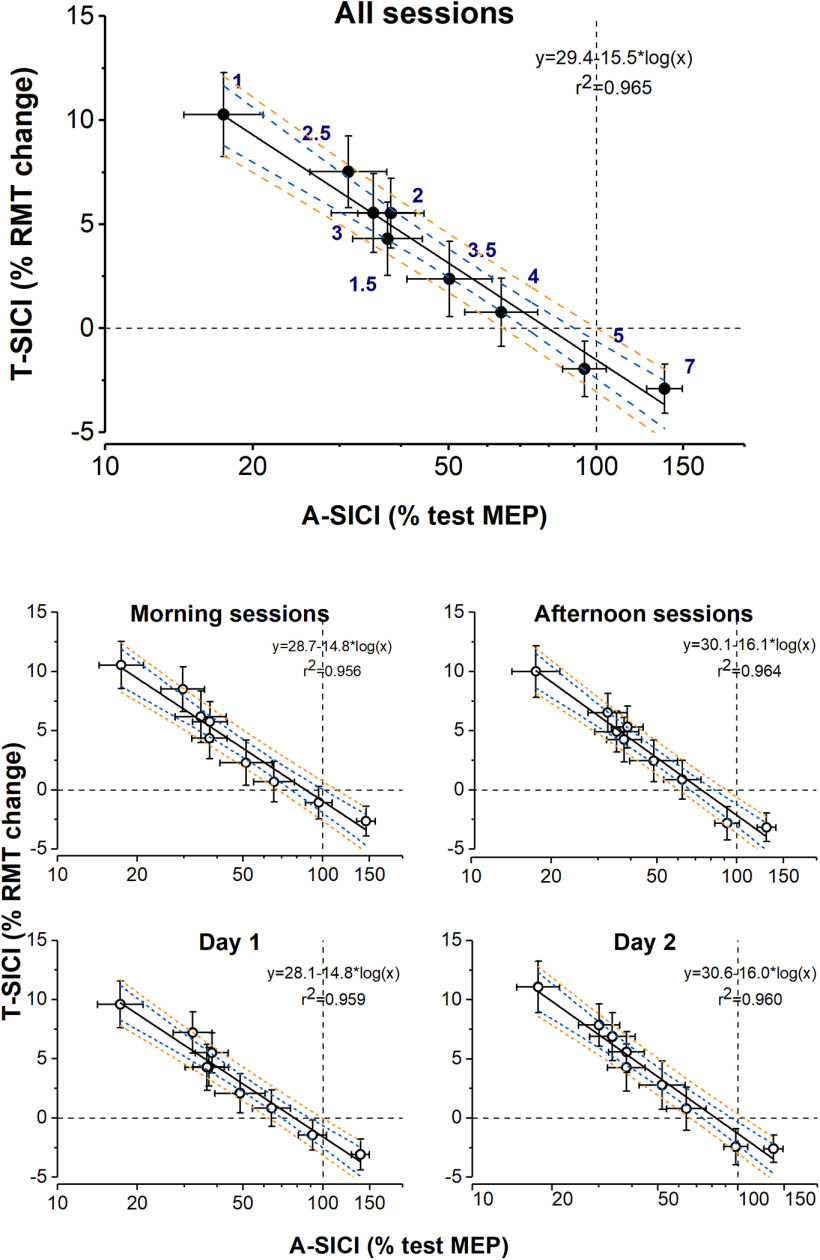
Relationship between mean group A-SICI and T-SICI curves. Group means of T-SICI (arithmetic) are plotted against A-SICI (geometric) at matching ISIs. *x*-axis (log_10_ scale): A-SICI obtained by cTMS. *y*-axis (linear scale): T-SICI obtained by TT-TMS. T-SICI and A-SICI group means were calculated by averaging all 18 individual subjects’ means across all measurements (all sessions), across measurements taken on the same time of day (morning and afternoon sessions), or the same experimental day (day 1 and day 2). Error bars represent SEM (±SEM for T-SICI, ×/÷SEM for A-SICI). A linear relationship (denoted by black solid line, blue dashed line 95% CI for regression line, and orange dashed line 95% prediction intervals for regression line) was observed between T-SICI and log-transformed A-SICI across ISIs (as indicated by navy numbers in the top panel), and was maintained throughout the experimental days as well as different time of the day.

**Figure 9.**
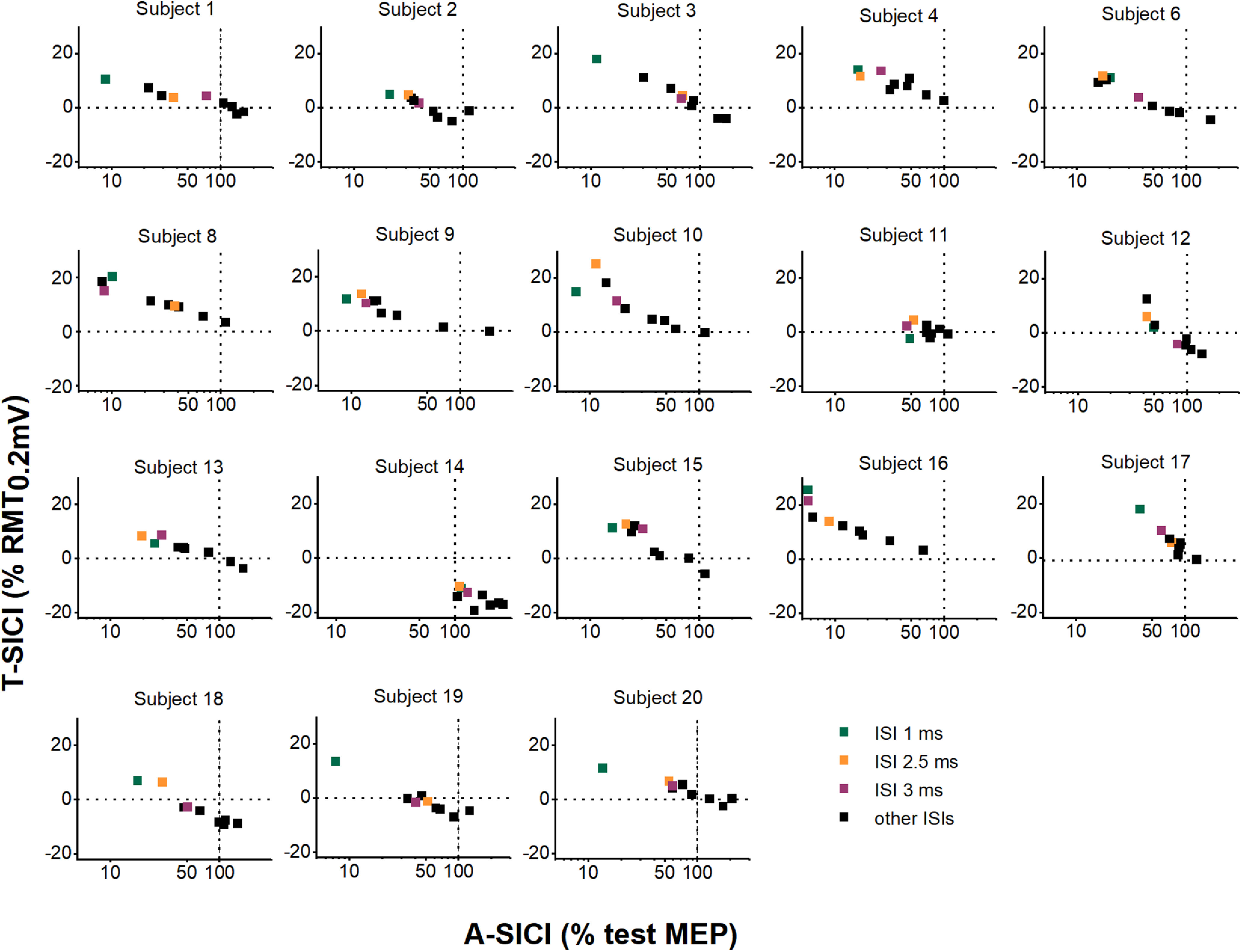
Relationship between A-SICI and T-SICI curves in individual subjects. Individual subject means of T-SICI (arithmetic) are plotted against A-SICI (geometric) at matching ISIs (calculated by averaging individual subjects’ means across all measurements). Black dashed lines indicate control conditions (0% RMT_0.2mV_ for T-SICI, 100% test MEP for A-SICI). *x*-axis (log_10_ scale): A-SICI obtained by cTMS. *y*-axis (linear scale): T-SICI obtained by TT-TMS. In many subjects, the relationship between log-A-SICI and T-SICI appeared to be linear or near-linear. However, in some there seemed to be a “floor effect” with cTMS that was overcome by TT-TMS (e.g., subject 12, subject 15); in others, no apparent correlation between the techniques was seen (e.g., subject 11, subject 14).

On a group level, a strong linear relationship between T-SICI and log-transformed A-SICI was observed across the whole range of tested ISIs, and this relationship was maintained throughout the experimental days as well as the time of the day (Fig. 8). However, there was considerable variability in this relationship in individual subjects with some discordance (i.e., one showing inhibition, another, facilitation) between the techniques at some ISIs (Fig. 9).

### Repeatability (intraobserver measurements)

Back-transformed CR for A-SICI_1ms_, A-SICI_2.5ms_, and A-SICI_3ms_ ranged from 2.6 (95% CI [2.1–4.6]) to 7.2 (95% CI [4.5–21.8]) on both days (Table 3). Thus, the difference between any two future examinations is estimated to be no greater than 2.6- to 7.2-fold difference on 95% of occasions for A-SICI_1ms_, A-SICI_2.5ms_, and A-SICI_3ms_. CR for T-SICI_1ms_, T-SICI_2.5ms_, and T-SICI_3ms_ ranged from 9.4% to 17.3% RMT_0.2mV_ on both days (Table 3), meaning that the absolute difference between any two future examinations is estimated to be no greater than 9.4−17.3% RMT_0.2mV_ on 95% of occasions for T-SICI_1ms_, T-SICI_2.5ms_, and T-SICI_3ms_.

**Table 3:** Repeatability of cTMS and TT-TMS parameters

			CR [95%CI]										
Method	TMS Parameter	Sample size (*n*)	Day 1						Day 2				
			Morning session			Afternoon session			Morning session			Afternoon session	
			CR	95%CI		CR	95%CI		CR	95%CI		CR	95%CI
cTMS	A-SICI_1ms_	17	3.0	[2.3–5.6]		6.0	[3.9–16.8]		3.8	[2.8–8.2]		5.7	[3.8–15.4]
	A-SICI_2.5ms_	17	3.4	[2.5–6.7]		7.2	[4.5–21.8]		2.6	[2.1–4.6]		3.2	[2.4–6.0]
	A-SICI_3ms_	17	4.4	[3.1–10.2]		6.0	[3.9–16.6]		2.7	[2.2–4.8]		4.1	[2.9–8.9]
	A-SICI_1-3.5ms_	17	2.0	[1.7–3.0]		4.9	[3.4–12.4]		2.2	[1.8–3.5]		2.5	[2.1–4.3]
	A-SICI_1-7ms_	17	1.9	[1.6–2.7]		2.5	[2.0–4.3]		2.0	[1.7–2.9]		1.8	[1.6–2.5]
	RMT_0.2mV_	17	5.2	[3.9–8.1]		6.4	[4.9–9.9]		5.2	[4.0–8.2]		5.0	[3.8–7.8]
	TS_1mV_	17	8.6	[6.6–13.5]		13.3	[10.1–20.7]		10.1	[7.8–15.8]		12.8	[9.8–20.0]
TT-TMS	T-SICI_1ms_	18	10.2	[7.8–15.7]		15.4	[11.9–23.8]		14.5	[11.2–22.3]		13.8	[10.7–21.3]
	T-SICI_2. ms_	18	9.4	[7.2–14.5]		13.5	[10.4–20.8]		12.9	[9.9–19.9]		17.3	[13.4–26.7]
	T-SICI_3ms_	18	13.5	[10.4–20.8]		13.5	[10.4–20.8]		9.9	[7.7–15.3]		12.2	[9.4–18.8]
	T-SICI_1-3.5ms_	18	8.0	[6.2–12.3]		8.5	[6.6–13.2]		7.9	[6.1–12.2]		9.0	[6.9–13.9]
	T-SICI_1-7ms_	18	6.9	[5.4–10.8]		7.6	[5.9–11.7]		6.4	[4.9–9.9]		8.4	[6.5–13.0]
	RMT_0.2mV_	18	6.5	[4.9–9.7]		5.1	[3.8–7.7]		5.0	[3.8–7.5]		10.7	[8.0–16.1]

For intraobserver (observer 1) measurements. Sample size *n* = 17 for cTMS and *n* = 18 for TT-TMS. SICI measured with the A-SICI protocol (A-SICI, a dimensionless ratio; see Materials and Methods, Statistical analysis, for further explanation) and the T-SICI protocol (T-SICI, in % RMT). The unit of RMT_0.2mV_ and TS_1mV_ is % MSO. CR for day 1 (morning and afternoon session 1) and day 2 (morning and afternoon session 2) were calculated to estimate repeatability.

CRs tended to be lower for averaged SICIs (SICI_1-3.5ms_ and SICI_1-7ms_) compared with the non-averaged SICIs (SICI_1ms_, SICI_2.5ms_, and SICI_3ms_). Also, CRs tended to be lower for morning SICI measurements compared with afternoon SICI measurements on both days. CRs for TS_1mV_ tended to be higher than for RMT_0.2mV_ in cTMS (Table 3).

### Intraday and interday reliability (intraobserver measurements)

**Figure 10.**
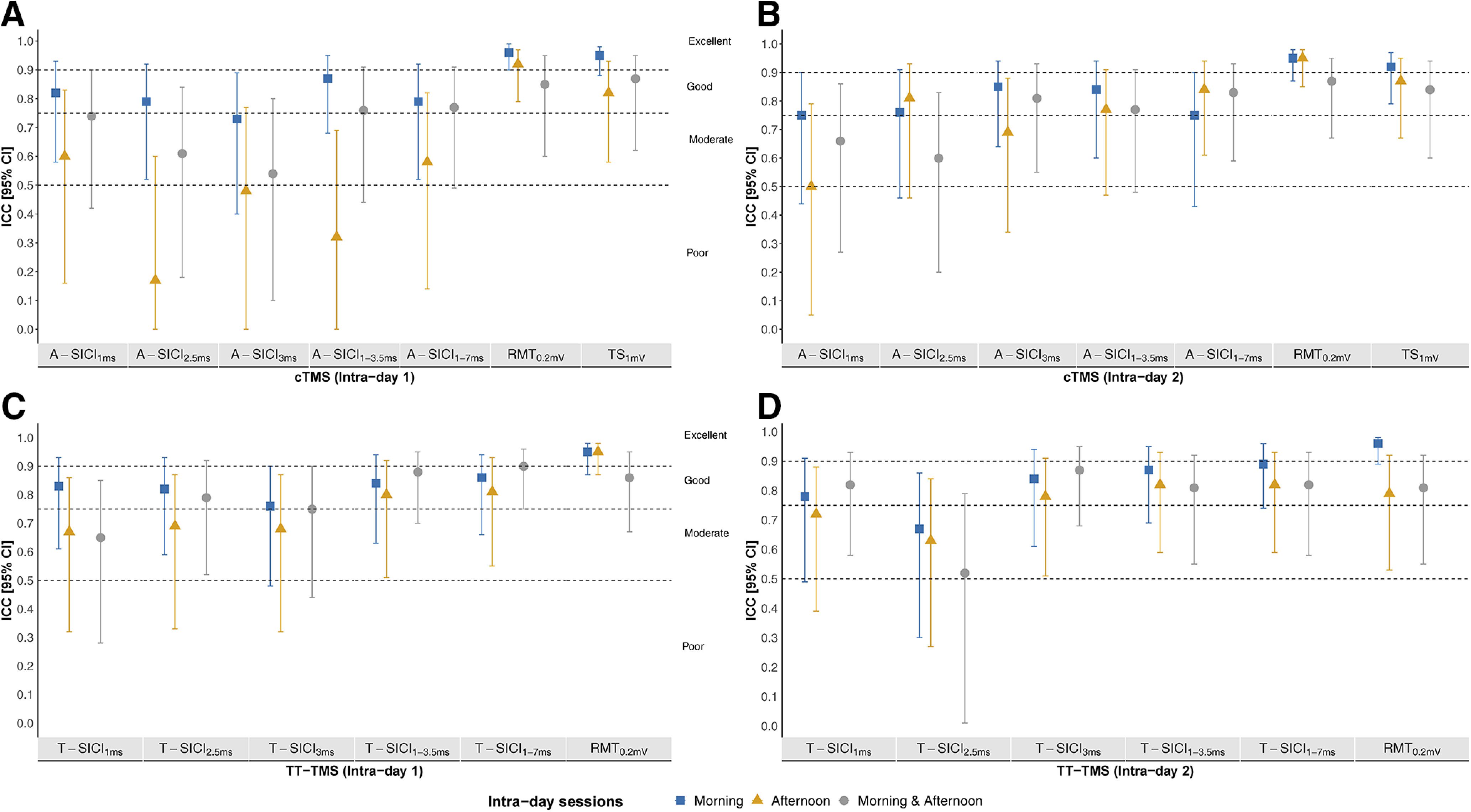
Intraday reliability of intraobserver cTMS and TT-TMS measurements. Intraobserver (observer 1) intraday reliability, estimated by ICC, of cTMS (***A***, ***B***) and TT-TMS (***C***, ***D***) parameters from day 1 (***A–C***) and day 2 (***B–D***). Sample size was *n* = 17 for cTMS and *n* = 18 for TT-TMS. *y*-axis: intraday ICC (2,1) with 95% CI for day 1 (***A–C***) and 2 (***B–D***). *x*-axis: cTMS and TT-TMS parameters. ICC intervals < 0.5, between 0.5 and 0.749, between 0.75 and 0.9, and intervals >0.9 indicated poor, moderate, good, and excellent reliability, respectively. Morning reliability (blue squares), afternoon reliability (yellow triangles), and morning and afternoon reliability (gray circles) are depicted from each measurement day. ICC calculations were based on measurements from the session of interest, i.e., morning session ICCs were based on the two morning measurements. Calculation of “morning and afternoon” reliability was based on data from the first morning measurement and first afternoon measurement.

On day 1, A-SICI morning reliability ranged from moderate to good, afternoon reliability ranged from poor-to-moderate, and intraday reliability was moderate-to-good (Fig. 10*A*). On day 2, A-SICI morning reliability was good, and afternoon and intraday reliability both ranged from moderate to good (Fig. 10*B*).

On day 1, T-SICI morning reliability was good, and afternoon and intraday reliability ranged from moderate to good (Fig. 10*C*). T-SICI reliability ranged from moderate to good on day 2 (Fig. 10*D*).

**Figure 11.**
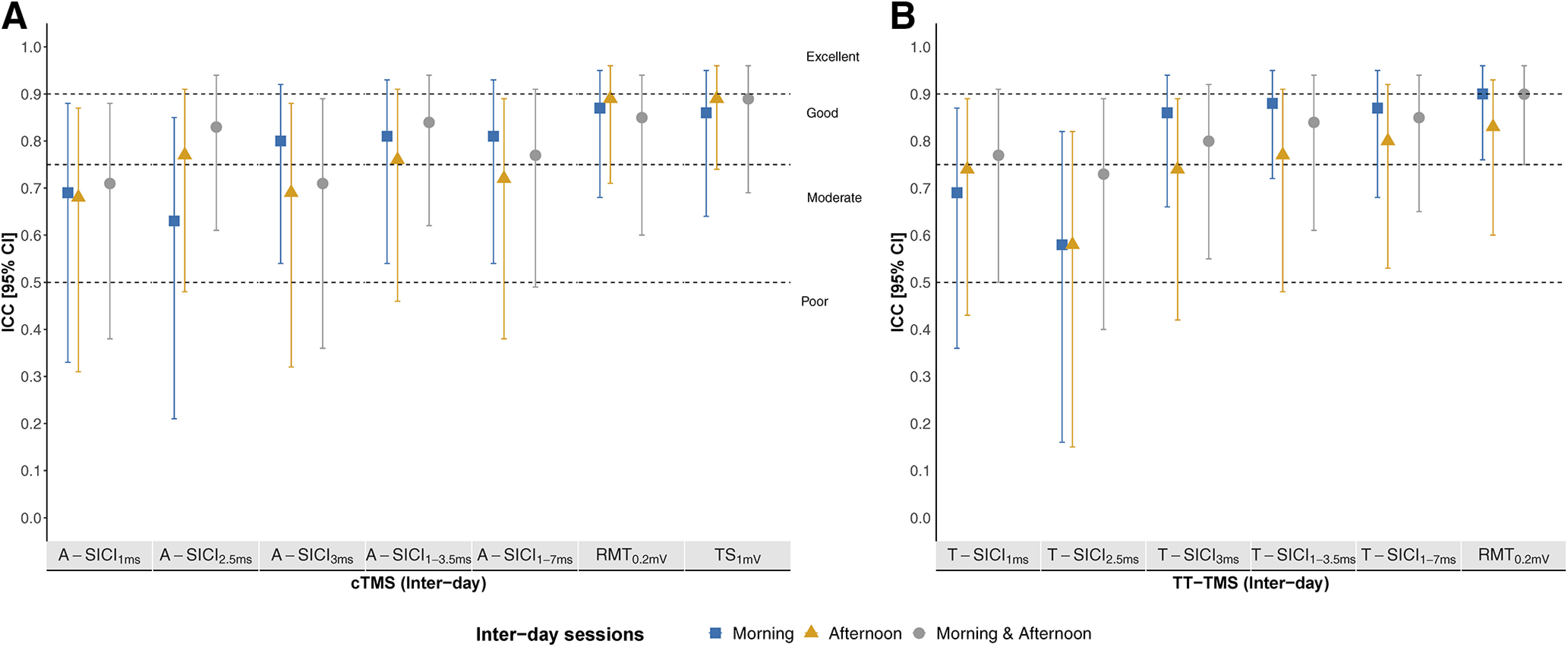
Interday reliability of intraobserver cTMS and TT-TMS measurements. Intraobserver (observer 1) interday reliability, estimated by ICC, of cTMS (***A***) and TT-TMS (***B***) parameters. Sample size was *n* = 17 for cTMS and *n* = 18 for TT-TMS. *y*-axis: interday ICC (2,1) with 95% CI for cTMS and TT-TMS. *x*-axis: cTMS and TT-TMS parameters. ICC intervals <0.5, between 0.5 and 0.749, between 0.75 and 0.9, and intervals > 0.9 indicated poor, moderate, good, and excellent reliability, respectively. Morning reliability (blue squares), afternoon reliability (yellow triangles), and morning and afternoon reliability (gray circles) are depicted. ICC was calculated to estimate interday reliability. Calculation of reliability was done by using data from the first measurement in each session from day 1 and day 2. Calculation of “morning and afternoon” (gray) reliability was based on data from the first morning measurement on day 1 and first afternoon measurement on day 2.

Interday reliability of all SICI measurements was moderate-to-good (Fig. 11). Intraday and interday reliability of all RMT_0.2mV_ and TS_1mV_ ranged from good to excellent (Figs. 10, 11).

### Reproducibility (intraobserver measurements)

None of the Bland–Altman plots for A-SICI measurements revealed ratios significantly different from 0, except for the A-SICI_2.5ms_ ratio $\frac{A-SICI_{2.5ms}examination7}{A-SICI_{2.5ms}examination8}$ = 0.73 (95% CI [0.56–0.94]) in the afternoon session on day 2. rmANOVA revealed that between-subject variation accounted for the largest part of total intersession variation, and that between-subject differences were significant for all TMS parameters. None of the TMS examinations differed significantly from another examination, except for RMT_0.2mV_ in cTMS (*F*_(4.13,66)_ = 2.53, *p* = 0.046), TS_1mV_ (*F*_(16,7)_ = 2.2, *p* = 0.038), and T-SICI_3ms_ (*F*_(17,7)_ = 2.49, *p* = 0.02). After correcting for multiple comparisons using Bonferroni correction, examination 4 differed significantly from examination 6 for T-SICI_3ms_ (*p* = 0.00035), and examination 1 differed significantly from examination 4 for cTMS TS_1mV_ (*p* = 0.00174), but none of the cTMS RMT_0.2mV_ examinations differed significantly from another.

### Interobserver reliability and reproducibility

Among interobserver reproducibility measurements, a significant difference was observed in the A-SICI_2.5ms_ ratio $\frac{{\text{A-SICI}}_{\text{2.5ms}}\text{ observer 2}}{{\text{A-SICI}}_{\text{2.5ms}}\text{ observer 1}}$ = 1.56 (95% CI [1.28–2.17]), *p* = 0.0103 (Table 4). None of the other comparisons (neither T-SICI differences, nor A-SICI ratios) were significantly different between observer 1 and observer 2.

**Table 4.** Description of interobserver measurements and reproducibility

Method	Parameter	Sample size	Number of measurements for each observer	Median [IQR]				Student’s paired *t* test		
				Observer 1			Observer 2		*t*	*p* value
cTMS	A-SICI_1ms_	18	18	16.6 [23.2]			15.9 [21.8]		2.11	0.45
	A-SICI_2.5ms_	18	18	25.3 [23.8]			40.8 [35.1]		2.11	**0.0103**
	A-SICI_3ms_	18	18	43.3 [33.1]			33.7 [35.4]		2.11	0.89
	A-SICI_1-3.5ms_	18	108	42.2 [27.1]^*b*^			31.5 [16.5]^*b*^		2.11	0.63
	A-SICI_1-7ms_	18	162	61.4 [38.2]^*b*^			44.3 [21.4]^*b*^		2.11	0.74
	RMT_0.2mV_	18	18	54.3 [8.4]			53.9 [8.8]		2.11	0.73
	TS_1mV_	18	18	65.9 [12.3]			64.5 [14.1]		2.11	0.46
										
				Mean (±SE)^a^	SD		Mean (±SE)^a^	SD		
			
TT-TMS	T-SICI_1ms_	18	18	9.4 (±2.01)	8.9		10.5 (±1.58)	6.7	2.11	0.49
	T-SICI_2.5ms_	18	18	9.4 (±2.49)	10.6		6.8 (±1.89)	8.0	2.11	0.28
	T-SICI_3ms_	18	18	5.5 (±2.43)	10.3		6.9 (±2.45)	10.4	2.11	0.47
	T-SICI_1-3.5ms_	18	108	6.2 (±1.53)	6.5		6.2 (±1.44)	6.1	2.11	0.98
	T-SICI_1-7ms_	18	162	3.1 (±1.65)	7.0		3.6 (±1.37)	5.8	2.12	0.82
	RMT_0.2mV_	18	18	54.5 (±1.96)	8.3		54.4 (±2.03)	8.6	2.11	0.84

Reproducibility of interobserver measurements of A-SICI and T-SICI. Sample size *n* = 18 for cTMS and TT-TMS for observer 2. Calculation of statistical parameters is based on TMS measurements from observer 2 (from one TMS measurement for each subject) and the corresponding examination (by session, first examination in the session) by observer 1. A significant difference between observers was seen in A-SICI_2.5ms_ (*p* = 0.0103) only. Differences were interpreted as significant at *p* < 0.05.

a Normally distributed data were arithmetically averaged to calculate mean (±SE) and SD.

b Point estimates for each subject were calculated as geometric means of their measurements. Data are displayed as medians with interquartile ranges [IQR] of subjects’ point estimates.

**Figure 12.**
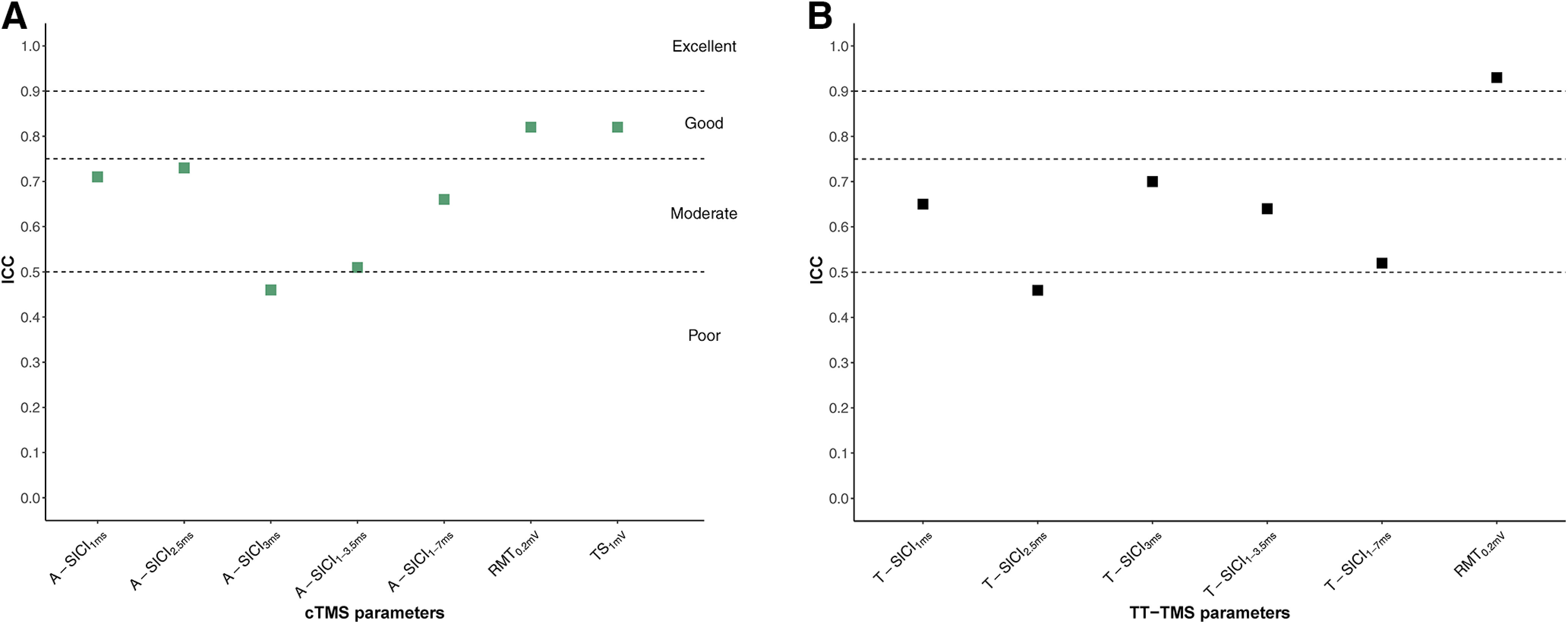
Interobserver reliability of cTMS and TT-TMS measurements. Interobserver (observer 1 and 2)reliability, estimated by ICC, of cTMS (***A***) and TT-TMS (***B***) parameters. Sample size was *n* = 18. *y*-axis: interobserver ICC (2,1). Calculation of statistical parameters is based on TMS measurements from observer 2 (from one TMS measurement for each subject) and the corresponding examination (by session, first examination in the session) by observer 1. *x*-axis: cTMS (green squares) and TT-TMS (black squares) parameters.

Interobserver reliability for SICIs ranged from poor-to-moderate, and good to excellent for RMT_0.2mV_ and TS_1mV_ (Fig. 12).

## Discussion

The present study elaborates multiple aspects of test-retest reliability of two emerging TMS protocols, including intraobserver and interobserver, intraday and interday reliability as well as diurnal influences on it. The present work extends current knowledge of the utility of these two novel SICI protocols.

### Comparability between the techniques

Earlier studies have compared parallel TT-TMS with cTMS for SICI with either multiple CS intensities and single ISI (Samusyte et al., 2018) or a single CS intensity and multiple ISIs (Tankisi et al., 2021a).

Despite some technical differences in the parallel threshold-tracking paradigm (such as tracking mode or maximum tracking step size), a good correlation between A-SICI and T-SICI measurements was observed in these studies both within and across tested SICI conditions, suggesting that both techniques reflect largely similar underlying physiological mechanisms, at least in healthy volunteers.

In the present study, a good correlation between SICI measurements obtained with the two protocols both at individual ISIs and averaged SICI was observed across subjects. Furthermore, a linear relationship between T-SICI and log-transformed A-SICI was found across the whole range of tested ISIs. On a group level, this relationship appeared to remain stable throughout the day or different experimental days. Nevertheless, considerable interindividual variability in A-SICI/T-SICI slopes was observed, which is similar to earlier findings comparing SICIs at multiple conditioning intensities (Samusyte et al., 2018). In several subjects (Fig. 9), a discrepancy between the methods was seen at some ISIs, which is probably reflected in the slightly different duration of inhibition in the group (1–3 ms for T-SICI vs 1–4 ms for A-SICI; Fig. 6). This cannot be explained by differences in CS intensity as it was set to 70% of tracked RMT_0.2mV_ for both techniques. Although it is common to adjust CS based on active motor threshold or conventional RMT of 0.05 mV (Rossini et al., 2015), RMT_0.2mV_ was used in the present study to ensure activation of comparable inhibitory neuron populations with both methods as SICI is known to vary depending on conditioning intensity (Kujirai et al., 1993; Vucic et al., 2009). However, because of the intrinsic differences in the techniques (predetermined test MEP size dependent on a constant test stimulus in cTMS versus predetermined conditioned MEP size resulting in varying test stimuli across ISIs in TT-TMS), the upper motor neuron populations tested by the two techniques may differ.

### Repeatability

CR can be used to estimate measurement error (Bartlett and Frost, 2008). In statistics, “measurement error” refers to the inherent continuous natural variation that occurs with repeated measurements of the same biological quantity in a subject. The measurement error may include natural biological variability in the subject and variability in the measurement method (Bland, 2015). Thus, measurement error does not refer to a mistake made during the examination, e.g., when an estimate is written down incorrectly (Bland, 2015).

One way to report measurement error is to estimate how much any two future measurements made on the same subject are expected to differ. This estimate may also be called “within-subject variation”: second measurements on the same subject are not expected to differ systematically from the first measurement, as this would indicate the values were not true replicates (Bland and Altman, 1999). Hence, the possibility of bias between measurements is excluded, and the measurement error depends only on the within-subject variation. Within-subject variation is therefore the same as the variation of the measurement error (Bartlett and Frost, 2008). CR estimates measurement error by quantifying the size of the differences between any two future measurements made on the same subject on 95% of occasions (Bland and Altman, 1999; Bartlett and Frost, 2008). Thus, the higher the CR, the higher the measurement error.

Measurement error in the present study may be ascribed to variation in coil placement and coil angling during the examination. The TMS coil was kept manually in place, and it was observed that even a slight change in coil angling by a few degrees or millimeters, either because of head movement by the subject or coil movement by the observer, could stimulate nearby muscles on the same hand, thereby possibly introducing measurement error. Likewise, the location of motor hotspot in the beginning of each measurement might have been subject to variation as well. The location was done manually, without a navigation system. It is possible that the exact same coil position and coil angling was not achieved in each consecutive examination, which might have contributed to the observed measurement error.

Measurement error in the present study may also be ascribed to biological variation in the subjects. Although the examinations were done in quick succession, the long duration of each session may have decreased subject alertness. A recent study demonstrated that spontaneously occurring fluctuations in alertness modulate cortical reactivity and MEP amplitude over relatively short durations in awake subjects (Noreika et al., 2020). If these findings can be extrapolated to the present study, then fluctuations in subject alertness may have contributed to biological variability, and thus measurement error in the present study. Furthermore, underlying oscillations in brain activity may affect TMS measurements (Zrenner et al., 2018; De Goede and Van Putten, 2019) and further contribute to the measurement error.

In the present study, the repeatability of RMT_0.2mV_ is in line with studies that used probabilistic methods to determine the conventional RMT with a 0.05 mV cutoff (Beaulieu et al., 2017). Meanwhile, the intraday CRs of RMT_0.2mV_ and TS_1mV_ are comparable to the previous study, which employed threshold-tracking and reported CRs of 5.5% and 10% MSO, respectively (Samusyte et al., 2018). The repeatability of A-SICI in the present study cannot be directly compared with previous studies which reported a wide range of CRs (17–147% test MEP; Fleming et al., 2012; Ngomo et al., 2012; Schambra et al., 2015; Samusyte et al., 2018). Because of non-normality, log-transformed A-SICI was used to calculate CRs. The back-transformed CRs are dimensionless and therefore it is not straightforward to apply them in clinical practice (Schambra et al., 2015). However, conceptually CRs are similar to Bland–Altman’s limits of agreement, which, if calculated with log-transformed values and then back-transformed, indicate a ratio to the value on the *x*-axis (Bland and Altman, 1999). Thus, measurement error of log-transformed values should be interpreted as a relative difference or fold-change between any two future measurements.

Meanwhile, the CRs for T-SICI were high when compared with their respective means. This shows that in an average subject with inhibition on the initial recording, some degree of facilitation could be observed with repeated testing, representing an expected variation (be it technical or biological). This is in keeping with previous reports of rather poor repeatability of T-SICI measurements in younger healthy volunteers (Matamala et al., 2018; Samusyte et al., 2018). The repeatability of SICI was improved by averaging multiple ISIs for both techniques, an observation which was also reported in another study (Matamala et al., 2018). This likely reflects the variation (biological and/or technical) of SICI versus ISI curves within subjects, which can be reduced by averaging across ISIs. Nevertheless, averaging is not sufficient to allow a confident use of these measurements for individual decision-making.

### Intraobserver and interobserver reliability

Overall, RMT_0.2mV_ and TS_1mV_ showed good-to-excellent reliability compared with poor-to-good intraobserver reliability, and poor-to-moderate interobserver reliability of paired-pulse measurements (Figs. 10-12). SICI_3ms_ and estimates averaged across ISIs tended to have higher interday ICCs, but no consistent pattern was observed for same-day recordings. None of the techniques demonstrated a superior reliability in the present study, in contrast to the previous report (Samusyte et al., 2018). This could be related to methodological differences, but it is also important to note that a direct comparison of ICCs between the studies cannot be made without taking into account the heterogeneity of the samples (Bartlett and Frost, 2008; Streiner and Norman, 2008; Beaulieu et al., 2017).

There was no significant bias in the TMS parameters obtained by different observers and the observer reliability of RMT_0.2mV_ and TS_1mV_ was similar. However, the interobserver ICCs for SICI were generally lower than intraobserver ICCs with both techniques, suggesting that longitudinal measurements should ideally be obtained by the same observer.

### Observer and intersession reproducibility

In general, intraobserver cTMS and TT-TMS measurements were reproducible and no significant differences between examinations were found, except for A-SICI_2.5ms_ (between examinations 7 and 8), and two intersessional differences for T-SICI_3ms_ (between examinations 4 and 6) and for cTMS TS_1mV_ (between examinations 1 and 4). Likewise, no significant interobserver differences were observed, except for the A-SICI_2.5ms_ between observer 1 and observer 2. This suggests that interobserver measurements were reproducible.

As A-SICI_2.5ms_ examination 7 by observer 1 was used in both A-SICI ratios that turned up statistically significant, it is likely that a bias was introduced in this examination, since it differed both from the same observer’s A-SICI_2.5ms_ examination 8 and from observer 2’s A-SICI_2.5ms_ examinations. It can only be speculated on, how and why a bias was introduced into only one parameter in just one of the examinations.

Considering the observed statistically significant differences seen with T-SICI_3ms_ and with cTMS TS_1mV_, it is unclear why exactly these two parameters differ significantly, when none of the other parameters from the same examinations differ. It is possible that even when controlling for family-wise error rate (FWER) using the Bonferroni procedure, one or both of the observed statistically significant differences were because of random chance, as a total of four “families” of comparisons were made (Motulsky, 2010).

### Is the time of day important?

There is limited data on the stability of TMS parameters throughout the day. No significant shift in RMT, MEP amplitude or conventional SICI has been previously observed in the awake state during the day (Koski et al., 2005; Lang et al., 2011; ter Braack et al., 2019). Our findings are also consistent with those of a previous study, which found no significant effect of time for SICI when tested at 9 A.M and 4.P.M (Doeltgen and Ridding, 2010). However, it has been proposed that SICI measurements obtained by TT-TMS may be more reliable if performed in the morning on different days compared with different times on the same day (Matamala et al., 2018). Indeed, it was observed in the present study that most SICI measurements obtained in the morning sessions tended to have better test-retest reliability indices (i.e., higher ICCs and/or lower CRs) compared with the afternoon sessions, both when measured on the same and different experimental days (Figs. 10, 11). Such a pattern was seen with both techniques, though more consistently with TT-TMS. Incidentally, it was observed in the present study that subjects found it more difficult to remain alert during the afternoon sessions. Although recently a nonlinear modulation of corticospinal excitability because of fluctuations in alertness has been described (Noreika et al., 2020), it is unclear whether this could have contributed to the increased variability in the afternoon in the present study. The intraday and interday reliability of SICI was largely comparable in the present study.

### Strengths and limitations

Automated recording protocols allow the observer to concentrate on coil positioning and minimize observer bias, which is crucial for longitudinal assessments and multicentre studies. No considerable systematic bias in the SICI measurements across multiple ISIs obtained by the same or different observers was found in the present study, supporting the use of such protocols.

The numerous observer examinations are an important strength of the present study as it provides reliability data for different experimental and clinical scenarios: (1) the “immediate” reliability when measurements are repeated in a quick succession (e.g., in studies of interventions with short-lasting effects or a repetition of a test to improve diagnostic certainty in clinics); (2) intraday reliability (e.g., interventional studies in which the effects are measured over the course of the day); (3) interday reliability (e.g., longitudinal assessments in clinical trials).

Although SICI reliability tended to be better in the morning than in the afternoon sessions, these findings were not statistically significant because of broad and overlapping 95% CI. This could be explained by a relatively small sample (Rankin and Stokes, 1998; Bonett, 2002). However, improved precision would require much larger samples (Bonett, 2002), which may not be practical given that ICCs cannot be easily generalized between different samples or populations with different variances (e.g., from healthy volunteers to patients).

While in many fields CRs and ICCs are used to define measurement error, a distinction between technical and biological variability cannot be made for TMS measurements. For example, the increased measurement error in the afternoon may be related to fluctuations in both the subject’s state and in the observer’s vigilance. Further studies using a robotic arm for coil positioning and thus eliminating the observer factor would shed more light on the biological variability of cortical excitability.

In addition to the different ISIs, SICI ISI averages of 1–3.5 and 1–7 ms were analyzed for consistency with earlier studies (Matamala et al., 2018; Menon et al., 2018; Ørskov et al., 2021; Tankisi et al., 2021a). Given the interindividual and intraindividual variability of SICI versus ISI curves, average measures may provide a more reliable parameter. Indeed, SICI averaged across ISIs of 1–7 ms has shown the best reproducibility (Matamala et al., 2018) and diagnostic utility for ALS in earlier studies with serial tracking (Menon et al., 2015), although some overlap with intracortical facilitation is likely reflected in this measure. Another SICI variable, an average across ISIs of 1–3.5 ms, has earlier been reported (Ørskov et al., 2021; Tankisi et al., 2021a). It represents intervals with maximum inhibition. However, one should remember that SICI at different ISIs have different underlying physiological mechanisms related to the refractory period, extrasynaptic and synaptic inhibition and overlap with short interval intracortical facilitation (Ziemann et al., 1996; Peurala et al., 2008; Stagg et al., 2011).

The potential differences as well as advantages and disadvantages of the two techniques have been discussed earlier (Samusyte et al., 2018). Briefly, threshold-tracking may allow a better evaluation of the full inhibitory potential as it overcomes the “floor effect” seen with cTMS. Meanwhile, A-SICI may be more suitable if one is interested in a particular subset of motor neurons. Threshold-tracking protocols can be preferred when the MEP amplitude is low, since in these conditions conventional method will be difficult to perform successfully. In contrast, in subjects with high RMT, the stimulator power may not be sufficient to capture full inhibition with threshold-tracking. As conventional and threshold-tracking protocols may potentially examine different neuron pools in healthy subjects (Samusyte et al., 2018) and patients (Tankisi et al., 2021b), they may have different sensitivity in pathologic conditions or respond differently to drugs. Future head-to-head comparisons in patient populations and interventional studies are warranted.

In conclusion, good correlations between SICI measurements obtained by cTMS and TT-TMS across a full range of ISIs was observed. The two techniques showed similar test-retest reliability profiles in healthy subjects with poor repeatability on the individual level, and satisfactory reliability on the group level. This suggests that the two automated SICI protocols may be reliably employed in research studies, but should at this moment be used with caution for individual decision-making in clinical settings. Further studies exploring reliability in different disease cohorts, such as motor neuron diseases or stroke, are warranted to investigate the diagnostic and clinical utility of the two automated SICI protocols.

## Synthesis

Reviewing Editor: Karen Davis, Krembil Research Institute and University of Toronto Decisions are customarily a result of the Reviewing Editor and the peer reviewers coming together and discussing their recommendations until a consensus is reached. When revisions are invited, a fact-based synthesis statement explaining their decision and outlining what is needed to prepare a revision will be listed below. The following reviewer(s) agreed to reveal their identity: Zhen Ni.

The present study compared a threshold-tracking protocol and a conventional protocol that both were used for measuring short-interval intracortical inhibition (SICI). Inter-sessional and inter-observer reliability were discussed with data obtained in four sessions performed at different times. It was found that the two protocols had no significant bias across sessions and had a comparable test-retest reliability profile. This study focused on an important topic in the field and was well performed. I only have a few comments. These are related to further discussion from a physiological viewpoint in addition to the most of current version of discussion from a technical viewpoint.

1, A major point is that it is not described very clearly why the topic is important in the field. I understand that the authors tried to explain it at the last part of introduction. However, please further discuss it from a physiological viewpoint.

2, In addition to Figure 1 legend, it would be helpful for the readers to better understand the experimental setup if four examinations are mentioned explicitly in an early paragraph (study design).

3, It should also be mentioned clearly that only the tracking technique was used for measuring the resting motor threshold in both protocols. I suppose a higher conditioning intensity was employed based on this technique. This point may also be discussed as SICI varies significantly with conditioning intensity.

4, The physiological meaning rather than the technical reason for plotting log MEP against stimulus intensity should be discussed.

5, It is not entirely clear why data with different interstimulus intervals (1-3.5 ms, 1-7 ms) were averaged. I think SICI at different interstimulus intervals are different. They are related to the mechanisms of refractory period, synaptic inhibition, short-interval intracortical facilitation, intracortical facilitation, etc. These should also be discussed.

6, The physiological meaning of coefficient of repeatability in this study should be discussed.

7, The reason why only a small portion of inter-sessional differences (both for intra-observer and inter-observer) were significant was not well explained.

8, It may be helpful for the end users if there is a short paragraph of discussion at which situations a threshold-tracking protocol may be used and at which situations a conventional protocol is recommended.

## Author Response

Dear Editor,

We would like to thank the editor and the reviewers for their careful reading of the manuscript and valuable comments. The responses to the questions are highlighted below in yellow font and, in the revision, deleted and inserted text is highlighted in yellow. We believe that the revisions have improved the manuscript and we hope that it is now acceptable for publication.

I have signed the License to Publish on behalf of all co-authors.

We look forward to your decision,

Yours sincerely,

Corresponding author (Name removed)

Synthesis Statement for Author (Required):

The present study compared a threshold-tracking protocol and a conventional protocol that both were used for measuring short-interval intracortical inhibition (SICI). Inter-sessional and inter-observer reliability were discussed with data obtained in four sessions performed at different times. It was found that the two protocols had no significant bias across sessions and had a comparable test-retest reliability profile. This study focused on an important topic in the field and was well performed. I only have a few comments. These are related to further discussion from a physiological viewpoint in addition to the most of current version of discussion from a technical viewpoint.

1, A major point is that it is not described very clearly why the topic is important in the field. I understand that the authors tried to explain it at the last part of introduction. However, please further discuss it from a physiological viewpoint.

Comment: We thank the Reviewer for this comment. The following has been added to the Introduction section:

"Earlier studies have shown that T-SICI may be used for diagnosing ALS and has been suggested as a biomarker (Vucic and Kiernan, 2006, 2008; Menon et al., 2015; Vucic and Rutkove, 2018)”.

"It was also proposed that due to a smaller between-subject variability among healthy individuals, A-SICI may be better at demonstrating more sensitive fora pathological loss of inhibition, which has been observed as an early feature of motor neuron disease (Vucic and Kiernan, 2006). A recent study has demonstrated that both techniques performed well at discriminating ALS patients from patient controls with T-SICI being most reduced before the upper motor neuron signs become apparent (Tankisi et al., 2021b). However, none of the studies that study did not assessed the test-retest reliability of the methods, which is important if an investigation is to be used for diagnostic purposes or interventional studies. A trend for improved reproducibility of T-SICI2.5ms has been reported (Samusyte et al., 2018), but it remains unclear whether this applies to other SICI ISIs. Further studies are needed to explore the utility of T-SICI and A-SICI in diagnostic decision making in ALS, but the comparison of these two methods´ reproducibility and reliability in healthy subjects should be investigated prior to implementation in clinics. Moreover, T-SICI and A-SICI may potentially investigate different motor neuron pools (Samusyte et al., 2018), and may therefore supplement each other in diagnostics and intervention studies.”

2, In addition to Figure 1 legend, it would be helpful for the readers to better understand the experimental setup if four examinations are mentioned explicitly in an early paragraph (study design).

Comment: We thank the Reviewer for this comment, and we agree that the four examinations should be mentioned more explicitly in the study design. We have revised the Method section (subsection Study design):

"To investigate intra-observer test-retest reliability, each subject was investigated by the same observer (Observer 1) on two separate days, five to seven days apart. On the first examination day, two sessions were conducted: a in four separate sessions on two separate days: two morning sessions (10.00- 11.30am) and an two afternoon sessions (1.00- 2.30pm). On the second examination day, a morning session (10.00- 11.30am) and an afternoon session (1.00- 2.30pm) were conducted again The two examination days were five to seven days apart (Figure 1). Each session consisted of four TMS examinations: two A-SICI examinations and two T-SICI examinations. On each examination day, each subject underwent four A-SICI examinations and four T-SICI examinations. Thus, eight A-SICI examinations and eight T-SICI examinations were conducted on each subject in total (Figure 1).

All examinations were executed at the same time of day for each subject. Each session consisted of four examinations, totalling 16 TMS examinations per subject. Each examination lasted on average 15 minutes, giving approximately one hour in total for each session.”

3, It should also be mentioned clearly that only the tracking technique was used for measuring the resting motor threshold in both protocols. I suppose a higher conditioning intensity was employed based on this technique. This point may also be discussed as SICI varies significantly with conditioning intensity.

Comment: We thank the Reviewer for this important point. We have amended the Method and Discussion sections accordingly (subsection Resting motor threshold). Our reply to comment 4 (see below) has been added to the same Method section (subsection Resting motor threshold), in the same paragraph:

Methods (subsection Resting motor threshold):

"This approach is based on work by Fisher et al. (Fisher et al., 2002), who found that the stimulus-response curve between the stimulus intensity for single pulse TMS and the MEP amplitude was approximately exponential over a hundredfold range of responses (...) Vucic et al. (Vucic and Kiernan, 2006) deduced that by setting a target MEP located in the midpoint of the log-linear interval, the variability in MEP amplitude translates to smaller changes in stimulus intensity, potentially overcoming this limitation.

The tracked RMT0.2mV estimate was then used to set the conditioning stimulus for both T-SICI and A-SICI protocols to ensure a comparable intensity between the techniques. The SICI protocol was then initiated (Figure 3).”

Discussion (subsection Comparability between techniques):

"In several subjects (Figure 9), a discrepancy between the methods was seen at some ISIs, which is probably reflected in the slightly different duration of inhibition in the group (1-3 ms for T-SICI vs 1-4 ms for A-SICI, Figure 6). This cannot be explained by differences in conditioning stimulus intensity as it was set to 70% of tracked RMT0.2mV for both techniques. Although it is common to adjust conditioning stimulus based on active motor threshold or conventional RMT of 0.05 mV (Rossini et al., 2015), RMT0.2mV was used in the present study to ensure activation of comparable inhibitory neuron populations with both methods as SICI is known to vary depending on conditioning intensity (Kujirai et al., 1993; Vucic et al., 2009). However, This could be due to the intrinsic differences in the techniques (predetermined test MEP size dependent on a constant test stimulus in cTMS versus predetermined conditioned MEP size resulting in varying test stimuli across ISIs in TT-TMS), the upper motor neuron populations tested by the two techniques may differ. which may result in different neuronal populations being tested.”

4, The physiological meaning rather than the technical reason for plotting log MEP against stimulus intensity should be discussed.

Comment: we thank the Reviewer for this comment. We have elaborated on the reason for plotting log MEP against stimulus intensity in the Method section (subsection Resting motor threshold):

” This approach is based on work by Fisher et al. (Fisher et al., 2002), who found that the relationship stimulus-response curve between the stimulus intensity for single pulse TMS and the MEP amplitude was approximately exponential over a hundredfold range of responses. When plotted on a logarithmic scale, the relationship between stimulus intensity and MEP amplitude of the MEP amplitude was approximately linear in an the interval from 0.02-2 mV. Target MEP was set at 0.2 mV, the midpoint in this log-linear interval (Fisher et al., 2002). Furthermore, the MEP distributions are often skewed (Nielsen, 1996), and the variability in MEP size, which could represent excitability fluctuations in cortical pyramidal cells and spinal motor neurons, may differ across stimulus intensities (Kiers et al., 1993). Thus, logarithmic transformation of amplitude data has been proposed to ensure normal distributions and to at least somewhat reduce the variability spread across the stimulus intensities (Nielsen, 1996; Goetz et al., 2014). Vucic et al. (Vucic et al., 2006) deduced that by setting a target MEP located in the midpoint of the log-linear interval, the variability in MEP amplitude translates to smaller changes in stimulus intensity, potentially overcoming these limitations.

The tracked RMT0.2mV estimate was then used to set the conditioning stimulus for both T-SICI and A-SICI protocols to ensure a comparable intensity between the techniques. The SICI protocol was then initiated (Figure 3). “

5, It is not entirely clear why data with different interstimulus intervals (1-3.5 ms, 1-7 ms) were averaged. I think SICI at different interstimulus intervals are different. They are related to the mechanisms of refractory period, synaptic inhibition, short-interval intracortical facilitation, intracortical facilitation, etc. These should also be discussed.

Comment: We agree with the Reviewer that different ISIs are related to the different mechanisms. We used these averaged intervals for consistency with the earlier threshold-tracking SICI studies (Matamala et al., 2018; Menon et al., 2018; Ørskov et al., 2021; Tankisi et al., 2021a). Additionally, in one study, the averaged SICI at 1-7ms showed the best reproducibility with serial tracking (Matamala et al., 2018).

We added the below sentence to the Discussion section (subsection Strengths and limitations):

” In addition to the different ISIs, we also analysed averages of 1-3.5 ms and 1-7 ms for consistency with the previous studies (Matamala et al., 2018; Menon et al., 2018; Ørskov et al., 2021; Tankisi et al., 2021a). Given the inter- and intraindividual variability of SICI vs ISI curves, average measures may provide a more reliable parameter. Indeed, SICI averaged across ISIs of 1-7 ms has shown the best reproducibility (Matamala et al., 2018) and diagnostic utility for ALS in previous studies with serial tracking (Menon et al., 2015), although some overlap with intracortical facilitation is likely reflected in this measure. Another SICI variable, an average across ISIs of 1-3.5 ms, has earlier been reported (Ørskov et al., 2021; Tankisi et al., 2021a). It represents intervals with maximum inhibition. However, one should remember that SICI at different ISIs have different underlying physiological mechanisms related to the refractory period, extra-synaptic and synaptic inhibition and overlap with short interval intracortical facilitation (Ziemann et al., 1996; Peurala et al., 2008; Stagg et al., 2011). “

6, The physiological meaning of coefficient of repeatability in this study should be discussed.

Comment: we agree with the Reviewer, that this should be discussed. A separate paragraph discussing the physiological meaning of coefficient of repeatability (CR) was added to the Discussion section (in subsection Repeatability). To assist the reader in the interpretation of CR, an elaboration of CR was added to both the Result section (subsection Repeatability (intra-observer measurements)) and the Discussion section (subsection Repeatability). In addition, a typo in Table 1 and a typo in the result section (subsection Repeatability (intra-observer measurements)) was corrected:

Result section (subsection Repeatability (intra-observer measurements)): elaboration of CR

"Back-transformed CR for A-SICI1ms, A-SICI2.5ms and A-SICI3ms ranged from 2.6 (95% CI [2.1-4.6]) to 6.0 7.2 (95% CI [4.5-21.8]) on both days (Table 3). Thus, the difference between any two future examinations is estimated to be no greater than 2.6-fold to 7.2-fold difference on 95% of occasions for A-SICI1ms, A-SICI2.5ms and A-SICI3ms. CR for T-SICI1ms, T-SICI2.5ms and T-SICI3ms ranged from 9.4% to 17.3% RMT0.2mV on both days (Table 3), meaning that the absolute difference between any two future examinations is estimated to be no greater than 9.4%-17.3% RMT0.2mV on 95% of occasions for T-SICI1ms, T-SICI2.5ms and T-SICI3ms.”

Result section (Table 1): in when to use:

"CR indicates how much two or more measurements made on the same subject will vary for on 95% of the subjects occasions (Bartlett and Frost, 2008).”

Discussion section (subsection Repeatability): elaboration of CR:

“However, conceptually CRs are similar to Bland-Altman’s limits of agreement, which, if calculated with log-transformed values and then back-transformed, indicate a ratio to the value on the x-axis (Bland and Altman, 1999). Thus, measurement error of log-transformed values should be interpreted as a relative difference or fold-change between any two future measurements. “

Discussion section (subsection Repeatability): discussion of CR:

"Coefficient of repeatability (CR) can be used to estimate measurement error (Bartlett and Frost, 2008). In statistics, “measurement error” refers to the inherent continuous natural variation that occurs with repeated measurements of the same biological quantity in a subject. The measurement error may include natural biological variability in the subject and variability in the measurement method (Bland, 2015). Thus, “measurement error” does not refer to a mistake made during the examination, e.g. when an estimate is written down incorrectly (Bland, 2015).

One way to report measurement error is to estimate how much any two future measurements made on the same subject are expected to differ. This estimate may also be called ‘within-subject variation’: Second measurements on the same subject are not expected to differ systematically from the first measurement, as this would indicate the values were not true replicates (Bland and Altman, 1999). Hence, the possibility of bias between measurements is excluded, and the measurement error depends only on the within-subject variation. Within-subject variation is therefore the same as the variation of the measurement error (Bartlett and Frost, 2008). CR estimates measurement error by quantifying the size of the differences between any two future measurements made on the same subject on 95% of occasions (Bland and Altman, 1999; Bartlett and Frost, 2008). Thus, the higher the CR, the higher the measurement error.

Measurement error in the present study may be ascribed to variation in coil placement and coil angling during the examination. The TMS coil was kept manually in place, and it was observed that even a slight change in coil angling by a few degrees or millimetres, either due to head movement by the subject or coil movement by the observer, could stimulate nearby muscles on the same hand, thereby possibly introducing measurement error. Likewise, the location of motor hotspot in the beginning of each measurement might have been subject to variation as well. The location was done manually, without a navigation system. It is possible that the exact same coil position and coil angling was not achieved in each consecutive examination, which might have contributed to the observed measurement error.

Measurement error in the present study may also be ascribed to biological variation in the subjects. Even though the examinations were done in quick succession, the long duration of each session may have decreased subject alertness. A recent study demonstrated, that spontaneously occurring fluctuations in alertness modulate cortical reactivity and MEP amplitude over relatively short durations in awake subjects (Noreika et al., 2020). If these findings can be extrapolated to the present study, then fluctuations in subject alertness may have contributed to biological variability, and thus, measurement error in the present study. Furthermore, underlying oscillations in brain activity may affect TMS measurements (Zrenner et al., 2018; De Goede and Van Putten, 2019) and further contribute to the measurement error.”

7, The reason why only a small portion of inter-sessional differences (both for intra-observer and inter-observer) were significant was not well explained.

Comment: We thank the reviewers for this important point. We have added a new subsection (titled Observer and inter-session reproducibility) in the Discussion section with an interpretation of inter-sessional differences. To assist the reader in the interpretation of the inter-observer and intra- and inter-sessional differences, the Method section (subsection statistical analysis) and the Result section (subsections “Reproducibility (intra-observer measurements) and Inter-observer reliability and reproducibility) were further elaborated. In addition, a typo (“day 1” was changed to “day 2”) was corrected in the Result section (subsection Reproducibility (intra-observer measurements):

Method section (subsection Statistical analysis):

"For normally distributed data, CR and Bland-Altman plots can be interpreted as the absolute difference between any two future replicate measurements estimated to be no greater than CR on 95% of occasions. However, this is not the case for CRs and Bland-Altman plots, which have been calculated with log-transformed data. Log-transformed CRs and Bland-Altman plots become dimensionless ratios. (...) Inter-observer statistical parameters were calculated by applying the first examination by Observer 1 in the corresponding session. If significant effects were identified (P<0.05), pairwise post hoc analysis with Bonferroni correction for multiple comparisons was applied. Inter-observer reliability was estimated by ICC.”

Result section (subsection Reproducibility (intra-observer measurements)): interpretation of intra- and inter-sessional differences and correction of typo:

"None of the Bland-Altman plots showed only significant differences, d, for A-SICI A-SICI2.5ms measurements revealed ratios significantly different from 0, except for the A-SICI2.5ms ratio (〖"A-SICI” 〗_"2.5ms” “ examination 7” )/(〖"A-SICI” 〗_"2.5ms” “ examination 8” )= 0.73 (95%CI [0.56-0.94]) in the afternoon session on day 21 (d = 0.73, 95% CI [0.56-0.94]). rmANOVA revealed that between-subject variation accounted for the largest part of total inter-session variation, and that between-subject differences were significant for all TMS parameters. None of the TMS examinations differed significantly from another examination were significantly different, except for RMT0.2mV in cTMS (F4.13,66=2.53, P=0.046), TS1mV (F16,7=2.2, P=0.038) and T-SICI3ms (F17,7=2.49, P= 0.02). After correcting for multiple comparisons using Bonferroni correction, showed significant differences between examination 4 & differed significantly from examination 6 for T-SICI3ms (P=0.00035), and between examination 1 differed significantly from examination& 4 for cTMS TS1mV (P=0.00174), but not none of the for cTMS RMT0.2mV examinations differed significantly from another. “

Result section (subsection Inter-observer reliability and reproducibility): Interpretation of inter-observer differences:

” Among inter-observer reproducibility measurements, a significant difference was observed in the A-SICI2.5ms ratio (〖"A-SICI” 〗_"2.5ms” “ observer 2” )/(〖"A-SICI” 〗_"2.5ms” “ observer 1” ) = 1.56 (95% CI [1.28-2.17]), P=0.0103, see Table 4. None of the other comparisons (neither T-SICI differences, nor A-SICI ratios) were significantly different between observer 1 and observer 2.

No significant inter-observer examination differences were observed (Student’;s paired t test), except for A-SICI2.5ms (P=0.0103, Table 4). Inter-observer reliability for SICIs ranged from poor-to-moderate, and good-to-excellent for RMT0.2mV and TS1mV (Figure 12). “

Discussion section: interpretation of inter-sessional differences (Observer and inter-session reproducibility):

” Observer and inter-session reproducibility

In general, intra-observer cTMS and TT-TMS measurements were reproducible and no significant differences between examinations were found, except for A-SICI2.5ms (between examinations 7 and 8), and two inter-sessional differences for T-SICI3ms (between examinations 4 and 6) and for cTMS TS1mV (between examinations 1 and 4). Likewise, no significant inter-observer differences were observed, except for the A-SICI2.5ms between Observer 1 and Observer 2. This suggests that inter-observer measurements were reproducible.

As A-SICI2.5ms examination 7 by Observer 1 was used in both A-SICI ratios that turned up statistically significant, it is likely that a bias was introduced in this examination, since it differed both from the same observer’;s A-SICI2.5ms examination 8 and from Observer 2’;s A-SICI2.5ms examinations. It can only be speculated upon, how and why a bias was introduced into only one parameter in just one of the examinations.

Considering the observed statistically significant differences seen with T-SICI3ms and with cTMS TS1mV, it is unclear why exactly these two parameters differ significantly, when none of the other parameters from the same examinations differ. It is possible that even when controlling for family-wise error rate (FWER) using the Bonferroni procedure, one or both of the observed statistically significant differences were due to random chance, as a total of four ‘;families’; of comparisons were made (Motulsky, 2010).”

8, It may be helpful for the end users if there is a short paragraph of discussion at which situations a threshold-tracking protocol may be used and at which situations a conventional protocol is recommended.

Comment: We agree with the Reviewer that such a paragraph is important. We added the paragraph below to the Discussion section (last paragraph in subsection Strengths and limitations):

” The potential differences as well as advantages and disadvantages of the two techniques have been discussed previously (Samusyte et al., 2018). Briefly, threshold-tracking may allow a better evaluation of the full inhibitory potential as it overcomes the ‘;floor effect’; seen with cTMS. Meanwhile, A-SICI may be more suitable if one is interested in a particular subset of motor neurons. Threshold-tracking protocols can be preferred when the MEP amplitude is low, since in these conditions conventional method will be difficult to perform successfully. In contrast, in subjects with high RMT, the stimulator power may not be sufficient to capture full inhibition with threshold-tracking. As conventional and threshold-tracking protocols may potentially examine different neuron pools in healthy subjects (Samusyte et al., 2018) and patients (Tankisi et al., 2021b), they may have different sensitivity in pathological conditions or respond differently to drugs. Future head-to-head comparisons in patient populations and interventional studies are warranted.”

References:

Bartlett JW, Frost C (2008) Reliability, repeatability and reproducibility: analysis of measurement errors in continuous variables. Ultrasound Obstet Gynecol 31:466-475.

Bland JM (2015) Clinical measurement. In: An introduction to medical statistics, 4th ed., pp 315-316. Oxford University Press.

Bland JM, Altman DG (1999) Measuring agreement in method comparison studies. Stat Methods Med Res 8:135-160.

De Goede AA, Van Putten MJAM (2019) Infraslow activity as a potential modulator of corticomotor excitability. J Neurophysiol 122:325-335.

Fisher RJ, Nakamura Y, Bestmann S, Rothwell JC, Bostock H (2002) Two phases of intracortical inhibition revealed by transcranial magnetic threshold tracking. Exp Brain Res 143:240-248.

Goetz SM, Luber B, Lisanby SH, Peterchev A V. (2014) A novel model incorporating two variability sources for describing motor evoked potentials. Brain Stimul 7:541-552.

Kiers L, Cros D, Chiappa KH, Fang J (1993) Variability of motor potentials evoked by transcranial magnetic stimulation. Electroencephalogr Clin Neurophysiol Evoked Potentials 89:415-423.

Kujirai T, Caramia MD, Rothwell JC, Day BL, Thompson PD, Ferbert A, Wroe S, Asselman P, Marsden CD (1993) Corticocortical inhibition in human motor cortex. J Physiol 471:501-519.

Matamala JM, Howells J, Dharmadasa T, Trinh T, Ma Y, Lera L, Vucic S, Burke D, Kiernan MC (2018) Inter-session reliability of short-interval intracortical inhibition measured by threshold tracking TMS. Neurosci Lett 674:18-23.

Menon P, Geevasinga N, Yiannikas C, Howells J, Kiernan MC, Vucic S (2015) Sensitivity and specificity of threshold tracking transcranial magnetic stimulation for diagnosis of amyotrophic lateral sclerosis: a prospective study. Lancet Neurol 14:478-484.

Menon P, Kiernan MC, Vucic S (2018) Cortical excitability varies across different muscles. J Neurophysiol 120:1397-1403.

Motulsky H (2010) Intuitive Biostatistics: A Nonmathematical Guide to Statistical Thinking, 2nd ed. 198 Madison Avenue, New York, New York 10016: Oxford University Press.

Nielsen JF (1996) Logarithmic distribution of amplitudes of compound muscle action potentials evoked by transcranial magnetic stimulation. J Clin Neurophysiol 13:423-434.

Noreika V, Kamke MR, Canales-Johnson A, Chennu S, Bekinschtein TA, Mattingley JB (2020) Alertness fluctuations when performing a task modulate cortical evoked responses to transcranial magnetic stimulation. Neuroimage 223:117305.

Ørskov S, Bostock H, Howells J, Pugdahl K, Fuglsang-Frederiksen A, Nielsen CSZ, Cengiz B, Samusyte G, Koltzenburg M, Tankisi H (2021) Comparison of figure-of-8 and circular coils for threshold tracking transcranial magnetic stimulation measurements. Neurophysiol Clin 51:153-160.

Peurala SH, M. Müller-Dahlhaus JF, Arai N, Ziemann U (2008) Interference of short-interval intracortical inhibition (SICI) and short-interval intracortical facilitation (SICF). Clin Neurophysiol 119:2291-2297.

Rossini PM et al. (2015) Non-invasive electrical and magnetic stimulation of the brain, spinal cord, roots and peripheral nerves: Basic principles and procedures for routine clinical and research application: An updated report from an I.F.C.N. Committee. Clin Neurophysiol 126:1071-1107.

Samusyte G, Bostock H, Rothwell J, Koltzenburg M (2018) Short-interval intracortical inhibition: Comparison between conventional and threshold-tracking techniques. Brain Stimul 11:806-817.

Stagg CJ, Bestmann S, Constantinescu AO, Moreno Moreno L, Allman C, Mekle R, Woolrich M, Near J, Johansen-Berg H, Rothwell JC (2011) Relationship between physiological measures of excitability and levels of glutamate and GABA in the human motor cortex. J Physiol 589:5845-5855.

Tankisi H, Cengiz B, Howells J, Samusyte G, Koltzenburg M, Bostock H (2021a) Short-interval intracortical inhibition as a function of inter-stimulus interval: Three methods compared. Brain Stimul 14:22-32.

Tankisi H, Nielsen CS-Z, Howells J, Cengiz B, Samusyte G, Koltzenburg M, Blicher JU, Møller AT, Pugdahl K, Fuglsang-Frederiksen A, de Carvalho M, Bostock H (2021b) Early diagnosis of amyotrophic lateral sclerosis by threshold tracking and conventional transcranial magnetic stimulation. Eur J Neurol.

Vucic S, Cheah BC, Krishnan A V., Burke D, Kiernan MC (2009) The effects of alterations in conditioning stimulus intensity on short interval intracortical inhibition. Brain Res 1273:39-47.

Vucic S, Howells J, Trevillion L, Kiernan MC (2006) Assessment of cortical excitability using threshold tracking techniques. Muscle Nerve 33:477-486.

Vucic S, Kiernan MC (2006) Novel threshold tracking techniques suggest that cortical hyperexcitability is an early feature of motor neuron disease. Brain 129:2436-2446.

Vucic S, Kiernan MC (2008) Cortical excitability testing distinguishes Kennedy’;s disease from amyotrophic lateral sclerosis. Clin Neurophysiol 119:1088-1096.

Vucic S, Rutkove SB (2018) Neurophysiological biomarkers in amyotrophic lateral sclerosis. Curr Opin Neurol 31:640-647.

Ziemann U, Lönnecker S, Steinhoff BJ, Paulus W (1996) The effect of lorazepam on the motor cortical excitability in man. Exp Brain Res 109:127-135.

Zrenner C, Desideri D, Belardinelli P, Ziemann U (2018) Real-time EEG-defined excitability states determine efficacy of TMS-induced plasticity in human motor cortex. Brain Stimul 11:374-389.

## References

[B1] Barnhart HX, Barboriak DP (2009) Applications of the repeatability of quantitative imaging biomarkers: a review of statistical analysis of repeat data sets. Transl Oncol 2:231–235. 10.1593/tlo.09268 19956383 PMC2781067

[B2] Bartlett JW, Frost C (2008) Reliability, repeatability and reproducibility: analysis of measurement errors in continuous variables. Ultrasound Obstet Gynecol 31:466–475. 10.1002/uog.5256 18306169

[B3] Beaulieu LD, Flamand VH, Massé-Alarie H, Schneider C (2017) Reliability and minimal detectable change of transcranial magnetic stimulation outcomes in healthy adults: a systematic review. Brain Stimul 10:196–213. 10.1016/j.brs.2016.12.008 28031148

[B4] Bland JM (2015) Clinical measurement. In: An introduction to medical statistics, Ed 4, pp 315–316. Oxford: Oxford University Press.

[B5] Bland JM, Altman D (1986) Statistical methods for assessing agreement between two methods of clinical measurement. Lancet 327:307–310. 10.1016/S0140-6736(86)90837-8 2868172

[B6] Bland JM, Altman DG (1999) Measuring agreement in method comparison studies. Stat Methods Med Res 8:135–160. 10.1177/096228029900800204 10501650

[B7] Bland JM, Altman DG (2003) Applying the right statistics: analyses of measurement studies. Ultrasound Obstet Gynecol 22:85–93. 10.1002/uog.122 12858311

[B8] Bonett DG (2002) Sample size requirements for estimating intraclass correlations with desired precision. Stat Med 21:1331–1335. 10.1002/sim.1108 12111881

[B9] Brown KE, Lohse KR, Mayer IMS, Strigaro G, Desikan M, Casula EP, Meunier S, Popa T, Lamy JC, Odish O, Leavitt BR, Durr A, Roos RAC, Tabrizi SJ, Rothwell JC, Boyd LA, Orth M (2017) The reliability of commonly used electrophysiology measures. Brain Stimul 10:1102–1111. 10.1016/j.brs.2017.07.011 28807846

[B10] De Goede AA, Van Putten MJAM (2019) Infraslow activity as a potential modulator of corticomotor excitability. J Neurophysiol 122:325–335. 10.1152/jn.00663.2018 31116669

[B11] de Vet HCW, Terwee CB, Knol DL, Bouter LM (2006) When to use agreement versus reliability measures. J Clin Epidemiol 59:1033–1039. 10.1016/j.jclinepi.2005.10.015 16980142

[B12] Doeltgen SH, Ridding MC (2010) Behavioural exposure and sleep do not modify corticospinal and intracortical excitability in the human motor system. Clin Neurophysiol 121:448–452. 10.1016/j.clinph.2009.11.085 20064743

[B13] Fisher RJ, Nakamura Y, Bestmann S, Rothwell JC, Bostock H (2002) Two phases of intracortical inhibition revealed by transcranial magnetic threshold tracking. Exp Brain Res 143:240–248. 10.1007/s00221-001-0988-2 11880900

[B14] Fleiss JL (1999) Reliability of measurement. In: The design and analysis of clinical experiments, pp 1–32. Hoboken: Wiley, Inc.

[B15] Fleming MK, Sorinola IO, Newham DJ, Roberts-Lewis SF, Bergmann JHM (2012) The effect of coil type and navigation on the reliability of transcranial magnetic stimulation. IEEE Trans Neural Syst Rehabil Eng 20:617–625. 10.1109/TNSRE.2012.2202692 22695363

[B16] Goetz SM, Luber B, Lisanby SH, Peterchev AV (2014) A novel model incorporating two variability sources for describing motor evoked potentials. Brain Stimul 7:541–552. 10.1016/j.brs.2014.03.002 24794287 PMC4108579

[B17] Groppa S, Oliviero A, Eisen A, Quartarone A, Cohen LG, Mall V, Kaelin-Lang A, Mima T, Rossi S, Thickbroom GW, Rossini PM, Ziemann U, Valls-Solé J, Siebner HR (2012) A practical guide to diagnostic transcranial magnetic stimulation: report of an IFCN committee. Clin Neurophysiol 123:858–882. 10.1016/j.clinph.2012.01.010 22349304 PMC4890546

[B18] Kiers L, Cros D, Chiappa KH, Fang J (1993) Variability of motor potentials evoked by transcranial magnetic stimulation. Electroencephalogr Clin Neurophysiol 89:415–423. 10.1016/0168-5597(93)90115-6 7507428

[B19] Koo TK, Li MY (2016) A guideline of selecting and reporting intraclass correlation coefficients for reliability research. J Chiropr Med 15:155–163. 10.1016/j.jcm.2016.02.012 27330520 PMC4913118

[B20] Koski L, Schrader LM, Wu AD, Stern JM (2005) Normative data on changes in transcranial magnetic stimulation measures over a ten hour period. Clin Neurophysiol 116:2099–2109. 10.1016/j.clinph.2005.06.006 16043397

[B21] Kujirai T, Caramia MD, Rothwell JC, Day BL, Thompson PD, Ferbert A, Wroe S, Asselman P, Marsden CD (1993) Corticocortical inhibition in human motor cortex. J Physiol 471:501–519. 10.1113/jphysiol.1993.sp019912 8120818 PMC1143973

[B22] Lang N, Rothkegel H, Reiber H, Hasan A, Sueske E, Tergau F, Ehrenreich H, Wuttke W, Paulus W (2011) Circadian modulation of GABA-mediated cortical inhibition. Cereb Cortex 21:2299–2306. 10.1093/cercor/bhr003 21350047

[B23] Matamala JM, Howells J, Dharmadasa T, Trinh T, Ma Y, Lera L, Vucic S, Burke D, Kiernan MC (2018) Inter-session reliability of short-interval intracortical inhibition measured by threshold tracking TMS. Neurosci Lett 674:18–23. 10.1016/j.neulet.2018.02.065 29501687

[B24] Menon P, Geevasinga N, Yiannikas C, Howells J, Kiernan MC, Vucic S (2015) Sensitivity and specificity of threshold tracking transcranial magnetic stimulation for diagnosis of amyotrophic lateral sclerosis: a prospective study. Lancet Neurol 14:478–484. 10.1016/S1474-4422(15)00014-9 25843898

[B25] Menon P, Kiernan MC, Vucic S (2018) Cortical excitability varies across different muscles. J Neurophysiol 120:1397–1403. 10.1152/jn.00148.2018 29975162

[B26] Motulsky H (2010) Intuitive biostatistics: a nonmathematical guide to statistical thinking, Ed 2. New York: Oxford University Press.

[B27] Ngomo S, Leonard G, Moffet H, Mercier C (2012) Comparison of transcranial magnetic stimulation measures obtained at rest and under active conditions and their reliability. J Neurosci Methods 205:65–71. 10.1016/j.jneumeth.2011.12.012 22227444

[B28] Nielsen JF (1996) Logarithmic distribution of amplitudes of compound muscle action potentials evoked by transcranial magnetic stimulation. J Clin Neurophysiol 13:423–434. 10.1097/00004691-199609000-00005 8897207

[B29] Noreika V, Kamke MR, Canales-Johnson A, Chennu S, Bekinschtein TA, Mattingley JB (2020) Alertness fluctuations when performing a task modulate cortical evoked responses to transcranial magnetic stimulation. Neuroimage 223:117305. 10.1016/j.neuroimage.2020.117305 32861789 PMC7762840

[B30] Ørskov S, Bostock H, Howells J, Pugdahl K, Fuglsang-Frederiksen A, Nielsen CSZ, Cengiz B, Samusyte G, Koltzenburg M, Tankisi H (2021) Comparison of figure-of-8 and circular coils for threshold tracking transcranial magnetic stimulation measurements. Neurophysiol Clin 51:153–160. 10.1016/j.neucli.2021.01.001 33468370

[B31] Peurala SH, Müller-Dahlhaus JF, Arai N, Ziemann U (2008) Interference of short-interval intracortical inhibition (SICI) and short-interval intracortical facilitation (SICF). Clin Neurophysiol 119:2291–2297. 10.1016/j.clinph.2008.05.031 18723394

[B32] Rankin G, Stokes M (1998) Reliability of assessment tools in rehabilitation: an illustration of appropriate statistical analyses. Clin Rehabil 12:187–199. 10.1191/026921598672178340 9688034

[B33] Roshan L, Paradiso GO, Chen R (2003) Two phases of short-interval intracortical inhibition. Exp Brain Res 151:330–337. 10.1007/s00221-003-1502-9 12802553

[B34] Rossi S, Hallett M, Rossini PM, Pascual-Leone A (2011) Screening questionnaire before TMS: an update. Clin Neurophysiol 122:1686. 10.1016/j.clinph.2010.12.037 21227747

[B35] Rossini PM, Burke D, Chen R, Cohen LG, Daskalakis Z, Di Iorio R, Di Lazzaro V, Ferreri F, Fitzgerald PB, George MS, Hallett M, Lefaucheur JP, Langguth B, Matsumoto H, Miniussi C, Nitsche MA, Pascual-Leone A, Paulus W, Rossi S, Rothwell JC, et al. (2015) Non-invasive electrical and magnetic stimulation of the brain, spinal cord, roots and peripheral nerves: basic principles and procedures for routine clinical and research application: an updated report from an I.F.C.N. committee. Clin Neurophysiol 126:1071–1107. 10.1016/j.clinph.2015.02.001 25797650 PMC6350257

[B36] Samusyte G, Bostock H, Rothwell J, Koltzenburg M (2018) Short-interval intracortical inhibition: comparison between conventional and threshold-tracking techniques. Brain Stimul 11:806–817. 10.1016/j.brs.2018.03.002 29573989 PMC6028741

[B37] Schambra HM, Ogden RT, Martínez-Hernández IE, Lin X, Chang YB, Rahman A, Edwards DJ, Krakauer JW (2015) The reliability of repeated TMS measures in older adults and in patients with subacute and chronic stroke. Front Cell Neurosci 9:335. 10.3389/fncel.2015.00335 26388729 PMC4555014

[B38] Stagg CJ, Bestmann S, Constantinescu AO, Moreno Moreno L, Allman C, Mekle R, Woolrich M, Near J, Johansen-Berg H, Rothwell JC (2011) Relationship between physiological measures of excitability and levels of glutamate and GABA in the human motor cortex. J Physiol 589:5845–5855. 10.1113/jphysiol.2011.216978 22005678 PMC3249054

[B39] Streiner DL, Norman GR (2008) Health measurement scales: a practical guide to their development and use. New York: Oxford University Press.

[B40] Tankisi H, Cengiz B, Howells J, Samusyte G, Koltzenburg M, Bostock H (2021a) Short-interval intracortical inhibition as a function of inter-stimulus interval: three methods compared. Brain Stimul 14:22–32. 10.1016/j.brs.2020.11.002 33166726

[B41] Tankisi H, Nielsen CS-Z, Howells J, Cengiz B, Samusyte G, Koltzenburg M, Blicher JU, Møller AT, Pugdahl K, Fuglsang-Frederiksen A, de Carvalho M, Bostock H (2021b) Early diagnosis of amyotrophic lateral sclerosis by threshold tracking and conventional transcranial magnetic stimulation. Eur J Neurol 28: 3030-3039. 10.1111/ene.15010 34233060 PMC9291110

[B42] ter Braack EM, de Goede AA, van Putten MJAM (2019) Resting motor threshold, MEP and TEP variability during daytime. Brain Topogr 32:17–27. 10.1007/s10548-018-0662-7 30019114 PMC6326963

[B43] Vucic S, Kiernan MC (2006) Novel threshold tracking techniques suggest that cortical hyperexcitability is an early feature of motor neuron disease. Brain 129:2436–2446. 10.1093/brain/awl172 16835248

[B44] Vucic S, Kiernan MC (2008) Cortical excitability testing distinguishes Kennedy’s disease from amyotrophic lateral sclerosis. Clin Neurophysiol 119:1088–1096. 10.1016/j.clinph.2008.01.011 18313980

[B45] Vucic S, Rutkove SB (2018) Neurophysiological biomarkers in amyotrophic lateral sclerosis. Curr Opin Neurol 31:640–647. 10.1097/WCO.0000000000000593 30080715

[B46] Vucic S, Howells J, Trevillion L, Kiernan MC (2006) Assessment of cortical excitability using threshold tracking techniques. Muscle Nerve 33:477–486. 10.1002/mus.20481 16315324

[B47] Vucic S, Cheah BC, Krishnan AV, Burke D, Kiernan MC (2009) The effects of alterations in conditioning stimulus intensity on short interval intracortical inhibition. Brain Res 1273:39–47. 10.1016/j.brainres.2009.03.043 19332031

[B48] Ziemann U, Lönnecker S, Steinhoff BJ, Paulus W (1996) The effect of lorazepam on the motor cortical excitability in man. Exp Brain Res 109:127–135. 10.1007/BF00228633 8740215

[B49] Zrenner C, Desideri D, Belardinelli P, Ziemann U (2018) Real-time EEG-defined excitability states determine efficacy of TMS-induced plasticity in human motor cortex. Brain Stimul 11:374–389. 10.1016/j.brs.2017.11.016 29191438

